# ATP-Binding and Hydrolysis in Inflammasome Activation

**DOI:** 10.3390/molecules25194572

**Published:** 2020-10-07

**Authors:** Christina F. Sandall, Bjoern K. Ziehr, Justin A. MacDonald

**Affiliations:** Department of Biochemistry & Molecular Biology, Cumming School of Medicine, University of Calgary, 3280 Hospital Drive NW, Calgary, AB T2N 4Z6, Canada; cfsandal@ucalgary.ca (C.F.S.); bjoern.ziehr@ucalgary.ca (B.K.Z.)

**Keywords:** inflammasome, NACHT domain, NOD-like receptor, NLR, NLRP, ATPase, molecular dynamic simulation, nucleotide

## Abstract

The prototypical model for NOD-like receptor (NLR) inflammasome assembly includes nucleotide-dependent activation of the NLR downstream of pathogen- or danger-associated molecular pattern (PAMP or DAMP) recognition, followed by nucleation of hetero-oligomeric platforms that lie upstream of inflammatory responses associated with innate immunity. As members of the STAND ATPases, the NLRs are generally thought to share a similar model of ATP-dependent activation and effect. However, recent observations have challenged this paradigm to reveal novel and complex biochemical processes to discern NLRs from other STAND proteins. In this review, we highlight past findings that identify the regulatory importance of conserved ATP-binding and hydrolysis motifs within the nucleotide-binding NACHT domain of NLRs and explore recent breakthroughs that generate connections between NLR protein structure and function. Indeed, newly deposited NLR structures for NLRC4 and NLRP3 have provided unique perspectives on the ATP-dependency of inflammasome activation. Novel molecular dynamic simulations of NLRP3 examined the active site of ADP- and ATP-bound models. The findings support distinctions in nucleotide-binding domain topology with occupancy of ATP or ADP that are in turn disseminated on to the global protein structure. Ultimately, studies continue to reveal how the ATP-binding and hydrolysis properties of NACHT domains in different NLRs integrate with signaling modules and binding partners to control innate immune responses at the molecular level.

## 1. The NLR Inflammasomes

### 1.1. Inflammasomes and Innate Immunity

Cells have developed an innate ability to respond to infectious pathogens as well as endogenous molecules that arise from cellular injury. A central driver for the innate immune system response to threats are inflammasomes, which are activated following detection of pattern- or damage-associated molecular patterns (PAMPs and DAMPs, respectively) by membrane or cytosolic pattern recognition receptors (PRRs) [1,2]. Inflammasomes are hetero-oligomeric protein complexes nucleated by cytosolic PRRs that maturate the pro-inflammatory precursor cytokines pro-IL-1β and pro-IL-18 [3,4,5]. Innate immune defences can also include a form of programmed cell death termed pyroptosis which occurs after inflammasome activation of inflammatory caspases, including human caspase-1, -4 and -5. Pyroptosis is a lytic and highly inflammatory form of programmed cell death that is characterized by swelling and membrane rupture; it is primarily actuated by caspase-1/-4 or -5-mediated cleavage of the pore-forming protein gasdermin D (GSDMD) [6,7,8,9]. The release of mature IL-1β and IL-18 as well as other endogenous DAMPs enables the activation and/or recruitment of macrophages, natural killer (NK) cells, and dendritic cells to generate a comprehensive immune response.

There are many PRRs, but the focus of this review is restricted to the cytosolic nucleotide-binding domain and leucine rich repeat-containing (NLR) gene family with 22 total members in the human genome [10]. Human NLR proteins diverge from other immune receptors and plant defense proteins by a central NACHT domain that displays nucleoside-triphosphatase (NTPase) activity. The evolutionarily-conserved NACHT domain derives its name from several of the proteins first identified to contain it, that is NAIP (NLP family apoptosis inhibitor protein), CIITA (class II major histocompatibility complex transactivator), HET-E (heterokaryon incompatibility protein from *Podospora anserina*) and TEP1 (telomerase protein component 1) [11]. Conservation of the NACHT domain in numerous intracellular ATPase proteins involved in innate immunity reflect the importance of this structural fold in protein function [12]. Sequence conservation places NLRs alongside the AP-ATPases (animal apoptosis regulators CED4/Apaf-1), plant disease resistance (R) proteins, bacterial AfsR-like transcription regulators, and putative NACHT NTPases identified in early green plants as members of the signal transduction ATPases with numerous domains (STAND) clade of the AAA+ ATPase superfamily of P-Loop NTPases (i.e., ATPases associated with various cellular activities) [13,14].

Collectively, STAND ATPases function to integrate, disseminate and amplify signals from a diverse range of stimuli through the activation of effector proteins. Their comparable large sizes and composite domain structure enable the nucleation of high molecular weight signaling complexes, thereby recruiting effectors and activating downstream signal transduction events. Their modular domain assemblies frequently combine variable N-terminal effector domains and C-terminal superstructure-forming sensor repeats with a central, conserved ATPase domain, supporting the importance of this domain in a family of signaling foci. While all subclades include crucial players in the signaling cascades of apoptosis and immunological defense mechanisms, members of the NACHT NTPases serve critical roles in both pro- and anti-apoptotic signal transduction (e.g., CARD4 and NAIP, respectively). The presence of STAND ATPases in all three major kingdoms of life indicate the early evolutionary emergence and critical importance of these molecules in immune function [13]. Of note, members of the related NACHT-NTPases and AP-ATPases do share closely conserved ATPase domains but are classified by their unrelated effector and sensor domains. This domain swapping and convergent evolution reflects the consequence of this mechanism of action in signaling and immune defense for all life forms.

**Figure 1:**
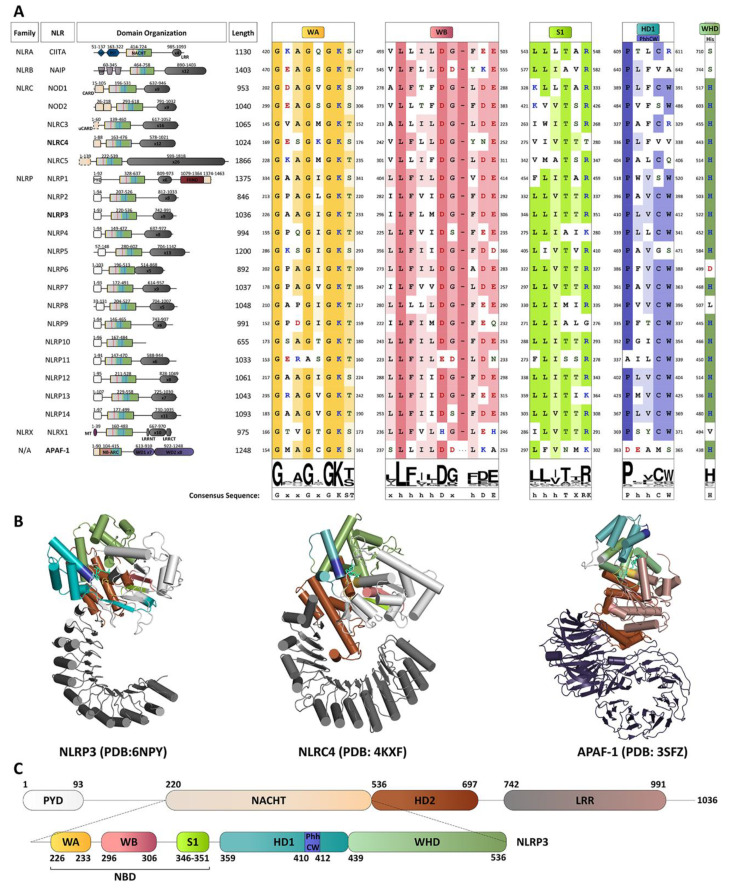
Characterization of NLRP3 and the NLR family. (**A**) Multiple sequence alignment of conserved features critical for catalytic activity in the 22 NLR proteins and APAF-1 NBD motifs. The NLR family members are subdivided based on the presence of one of four N-terminal effector domains: NLRAs with an acidic domain (AD), NLRBs with a Baculovirus IAP repeat (BIR), NLRCs with a caspase recruitment domain (CARD), or NLRPs with a pyrin domain (PYD). The naming system approved by the HUGO Gene Nomenclature Committee (HGNC) is used [10]. Domain identities and configuration for NLR proteins are displayed to scale with boundaries numbered as per UniProtKB accession numbers: CIITA, P33076; NAIP, Q13075; NOD1, Q9Y239; NOD2, Q9HC29; NLRC3, Q7RTR2; NLRC4, Q9NPP4; NLRC5, Q86WI3; NLRX1, Q86UT6; NLRP1, Q9C000; NLRP2, Q9NX02; NLRP3, Q96P20; NLRP4, Q96MN2; NLRP5, P59047; NLRP6, P59044; NLRP7, Q8WX94; NLRP8, Q86W28; NLRP9, Q7RTR0; NLRP10, Q86W26; NLRP11, P59045; NLRP12, P59046; NLRP13, Q86W25; and NLRP14, Q86W24. Multiple sequence alignments were created with Jalview [16] and MUSCLE [17] with default options and used to generate sequence conservation logos shown below each motif [18]. These indicate sequence conservation amongst NLRs and APAF-1, with the height of the stack indicating overall sequence conservation at that position in the alignment, while the height of letters within the stack indicates the relative frequency of each amino acid at that position. Amino acid class in the alignment is denoted by coloured font; where hydrophobic residues (A,I,L,M,F,W,V,C,G,P) are coloured in yellow; polar residues in green; polar (S,T,Y,N,Q) in green; acidic (D,E) in red; and basic (K,R,H) in blue. Conservation within each motif is denoted by background shading, where no color indicates <30% conservation, and increasing colour intensity indicates conservation of residue type from 30–60%, 60–80% or >80%. (**B**) Cartoon representation of NLR proteins NLRP3 and NLRC4 as well as APAF1, a close structural homolog. Domains and motifs are coloured as in (A). (**C**) Schematic representation of human NLRP3 showing important domains and motifs. The labelled domain boundaries were defined as per UniProtKB accession Q96P20-1. Important motifs within the NACHT domain were identified based on previous definitions. PYD, pyrin domain; NBD, nucleotide-binding domain; HD1, Helical domain 1; NACHT, ATPase domain named after its discovery in NAIP, CIITA, HET-E and TP1 apoptosis regulator proteins; WHD, Winged helix domain; HD2, Helical domain 2; WA, Walker A motif; WB, Walker B motif; S1, Sensor 1 motif; HD1: Helical domain 1; xVP, NLR xVP motif; LRR, Leucine rich repeat; AD, Acidic domain; PST, Proline-serine-threonine-rich domain; CARD, CAspase recruitment domain; uCARD, Untypical CARD domain; FIIND, domain with function to find; MT, Mitochondrial-targeting sequence; LRRNT, LRR N-terminal domain; LRRCT, LRR C-terminal domain; NB-ARC, Nucleotide-binding adaptor shared by APAF-1, R proteins, and CED-4; and WD, WD40 repeats. (For interpretation of the references to colour in this figure legend, the reader is referred to the Web version of this article.).

Accordingly, all NLRs (with the exception of NLRP10) contain a C-terminal leucine rich repeat (LRR) domain that serves a variety of roles, including auto-inhibition and ligand sensing in some cases (Figure 1A). A structural model of NLRC4 intimated that in the absence of danger signals, both the NACHT domain and the LRRs contribute to auto-inhibition of the receptor proteins, and the conservation of key interacting regions suggest the preservation of this mechanism amongst other NLRs [15]. The NLR proteins can be further sub-classified into five sub-groups based on the presence of different N-terminal domains: NLRA (acidic transactivating domain (AD)-containing; CIITA), NLRB (baculovirus inhibitor of apoptosis protein repeat (BIR)-containing; NAIP), NLRC (caspase activation and recruitment domain (CARD)-containing; NOD1, NOD2, and NLRC3-5), NLRP (pyrin domain (PYD)-containing; NLRP1-14), and NLRX (other domain containing; NLRX1) [10].

The prototypical model of inflammasome assembly includes nucleotide-dependent activation of the NLR downstream of PAMP or DAMP recognition. This event is followed by nucleation of hetero-oligomeric inflammasome platforms that lie upstream of the inflammatory responses associated with innate immunity [19,20]. Ultimately, the inflammasome provides a large supramolecular organizing center of enzymes and other regulatory proteins that provides a signal transduction scaffold for activation of pro-caspase-1 [21]. Full-length caspase-1 exists initially as a zymogen with two C-terminal subunits (small p10 and large p20 joined by an interdomain linker region) and an N-terminal CARD [22]. The assembled inflammasome elicits auto-processing and activation of caspase-1 which in turn triggers the maturation and secretion of pro-inflammatory mediators. In its most reductionist state, the inflammasome complex contains a sensor of DAMPs/PAMPs (e.g., NLR), an adaptor protein that possesses a CARD (e.g., ASC; apoptosis-associated, speck-like protein containing a CARD), and an effector enzyme (e.g., caspase-1 that is recruited to inflammasomes via a CARD). Homotypic CARD-CARD and PYD-PYD interactions between components are required for assembly of the inflammasome scaffold.

Notably, dysregulation of inflammasome activation can contribute to a multitude of inflammatory diseases, as well as to auto-immune disorders, cardiometabolic diseases, microbiome homeostasis, cancer, neurological disorders and more. The substantive role of NLRs in disease has been reviewed extensively so is not the focus of this article (see topical reviews: [7,23,24,25,26,27,28,29]. Due to the significance of NLRs in disease and their characterization as critical drug targets to modulate inflammation, the biochemical and structural interrogation of NLRs has emerged as an area of vigorous investigation. In 2002, NLRP1 was the first NLR receptor reported to oligomerize and assemble an inflammasome in association with ASC and procaspase-1 [30]. Now, well-established inflammasome complexes include the NLRC4, NLRP1, NLRP3, AIM2 and PYRIN signaling platforms.

Many inflammasome complexes are thought to share a similar model of ATP-dependent activation and effect. The NLRC proteins contain a CARD-NACHT-LRR domain organization, whereas a PYD-NACHT-LRR construct is most conserved for the NLRPs (Figure 1A). Exceptions to this structural domain organization include NLRP1 which has an additional C-terminal Function to Find domain (FIIND) adjacent to a CARD, and NLRP10 which lacks an LRR. As the prototype, the assembly of the NLRP3 inflammasome requires ASC to bridge to the NLRP3 receptor (via homotypic PYD-PYD interactions) and to caspase-1 (via homotypic CARD-CARD interactions) [31]. For NLRP1, the autoproteolytic cleavage of FIIND leads to release of a bioactive C-terminal CARD-containing fragment that can recruit caspase-1 directly and is sufficient for inflammasome activation [32,33,34]. CARD domains enable the direct interaction with pro-caspase-1 via homotypic CARD-CARD interactions [35,36,37], so NLRCs (and NLRP1) can induce inflammasome assembly in an ASC-independent manner. Despite this, ASC is still found to be necessary for efficient production of bioactive cytokines from inflammasomes that lack the ability to form homotypic PYD-PYD interactions. Finally, the assembly of AIM2 and PYRIN inflammasomes are also dependent upon ASC. PYRIN and AIM2 are members of the TRIM and absent in melanoma 2 (AIM2)-like receptor (ALR) gene families, respectively; however, these two inflammasomes do not contain an NTP-binding NACHT domain, so they are not discussed further herein [21,38,39]. There are also emergent investigations of other NLR receptors that assemble inflammasome signaling platforms; these include NLRP2, NLRP6, NLRP7, NLRP12, and NLRC5. The molecular assembly processes for these inflammasomes are less well characterized, and there is a lack of structural data, relative to those available for NLRC4, NLRP1 and NLRP3 [20,40].

All NLRs are thought to engage an active conformation by binding a nucleotide, and many have been shown to be dependent upon NTPase activity for proper function [15,41,42,43,44,45]. Conservation of key functional modules within the family suggest that certain mechanisms of action could be shared amongst NLR member proteins, and these structural and functional linkages will be reviewed in upcoming sections. With NTP binding, the NACHT domain is presumed to undergo a conformational shift into the active state; however, the specific relationship between NTP binding and hydrolysis and the NLR protein conformational change that leads to inflammasome assembly is not fully understood [46,47]. For the NLRP members, activation following NTP-binding and/or hydrolysis provides the opportunity for homotypic PYD-PYD binding between the NLRP and adaptor protein ASC, which then recruits pro-caspase-1 via CARD-CARD interactions.

### 1.2. Selective Tissue Expression Profiles

The gene expression level of NLRs can be estimated from the average transcript per million (TPM) value annotated for individual samples obtained from a particular human tissue or cell type. In this case, a consensus dataset generated after application of a normalization pipeline to three transcriptomic repositories (i.e., Human Protein Atlas, HPA [48]; genome-based tissue expression, GTEx [49]; and functional annotation of the mammalian genome project, FANTOM5 [50]). A gene is associated with enhanced expression for a given tissue when a significantly higher mRNA level is identified in a particular tissue compared to the average level in all other tissues combined. Although many of the 22 NLRs are expressed in low amounts across many different tissue types, nine NLRs have enhanced mRNA expression in lymphoid tissues, eight NLR transcripts are enhanced in blood/immune cell types (four are found to be elevated in both lymphoid and blood cells), and three have no specific tissue enhancement. Of the eight NLRs that have enhanced expression in blood immune cell types, five are specific to granulocytes (basophils, eosinophils and neutrophils). NLRP4 and NLRP11 have enhanced expression in B-cells; however, the absolute magnitude is relatively low. Finally, NLRP6 is expressed exclusively in the gastrointestinal tract (i.e., duodenum, small intestine and colon). It is worth emphasizing that expression does not imply translation, and protein level orchestration of the innate immune system is critical since the system needs to engage quickly in response to DAMPs and PAMPS. In fact, one interesting facet of regulation was observed for Nlrp6 in hepatocytes, in which transcripts were found to be retained in the nucleus, thus buffering gene expression noise emanating from transcriptional bursts [51].

### 1.3. Epigenetic Programming and Innate Immune Memory

Following microbial infection, innate immune cells may undergo long term epigenetic reprogramming into hyperactive states and thereby increase their resistance in reinfection. This biological response has been termed “trained immunity” or “innate immune memory” [52,53]. Macrophages, NK cells, and monocytes are the most studied cells found to exhibit innate immune memory, but any bone marrow myeloid progenitor cell can be subjected to epigenetic programming for the establishment of innate immune memory.

On exposure to a primary stimulus (e.g., *Candida albicans*, BCG vaccine, muramyl dipeptide), innate immune cells undergo changes in histone acetylation, histone methylation and DNA methylation to modulate a variety of transcriptional and inflammatory pathways [54,55]. Additionally, trained cells contain long-lasting regulatory miRNAs that are not present (or significantly supressed) in resting cells. Secondary exposure to trained cells often results in expression hundreds of times greater than in untrained controls. This is driven largely by stimulation-responsive transcription factors (e.g., NFκB, AP-1, STAT family members). These effects last weeks, months and even years, with methylation events outlasting short term acetylations. Many of the mechanisms whereby the epigenetic regulators are specifically activated to induce innate immune memory remain unknown. Interestingly, however, there is a growing body of evidence linking metabolic disruption to the epigenetic changes associated with trained immunity. One example was provided by a study which found that trained immunity induced by β-glucan was dependent on an Akt/mTOR/HIF-1α pathway during transition to glycolysis from OX-PHOS in macrophages following activation [56]. Additionally, several groups have shown that oxidized low-density lipoprotein (oxLDL) is capable of driving innate immune memory. NLRP3 is likely to be the molecular effector since mTOR, HIF-1α and oxLDL are all known activators of this inflammasome [57].

Recently, a NLRP3 dependent *sterile* inflammatory model of trained immunity was described in an experimental model of atherosclerosis with *LdlR*^−/−^ mice subjected to a Western diet [58]. After 4 weeks on a high saturated-fat diet, mice had elevated levels of inflammatory cytokines, were hypersensitive to LPS stimulation, and had granulocyte myeloid progenitors (GMPs) that displayed long-lived epigenetic modifications consistent with innate immune memory. Of the transcriptional changes observed, genes associated with metabolic processes as well as the positive regulation of cell death, transcription and proliferation were upregulated when compared to animals on a customary chow diet. In the absence of NLRP3 peripheral monocytosis did not occur, and most of the transcriptional changes associated with consumption of Western diet were not observed. Overall, NLRP3 appeared necessary for granulocyte myeloid progenitor proliferation, hypersensitivity to LPS stimulation, and most of the sustained transcriptional changes caused by a Western diet in this mouse model for the early, initiation phase of atherosclerosis, implicating the importance of NLRP3 inflammasomes for epigenetic modifications during induction of innate immune memory. Likely, the broad cytokine-dependent inflammation caused by activated NLRP3 inflammasomes promotes complex epigenetic reprogramming that ultimately regulate transcription and influence trained immunity. Consequentially, inflammation-responsive transcriptional regulators are left inactivated and the immunity training does not occur in the absence of NLRP3.

### 1.4. NLR Links between the Innate and Adaptive Immune Systems

Inflammasomes play an important role in activating the adaptive immune system [59,60]. When functioning properly, the purpose of the innate immune response is to immediately prevent the spread of pathogens throughout the body, and to initiate the second line of defense, the adaptive immune response (e.g., T cells). This is accomplished primarily from secretion of IL-1 cytokine family members (i.e., IL-1β, IL-18, and IL-1α). Caspase-1 activation also facilitate secretion of high mobility group protein (HMGB)-1, which in conjunction with IL-1 family cytokines influences T cell immunity via Th17 differentiation, T cell proliferation, and Th2 cell polarization. Moreover, IL-18 can also act through IL-1R and IL-18R and alter interferon (INF)-γ production from Th1 cells and NK cells.

A number of NLRs have been implicated in activating the adaptive immune system following infection through cytokine signalling. There is preliminary data linking NLRP1, NLRP6, NLRP10 and NLRP12 to the activation of the adaptive immune system; however, more comprehensive investigations are required to validate these connections. Definite links have been established for the importance NLRP3 and NLRC4 inflammasomes in signalling to engage proper adaptive immune responses to bacterial, viral and fungal pathogens. The NLRC4 inflammasome produces IL-18 which subsequently increases IFN-γ secretion from memory CD8+ T cells and IL-17 producing T cells during *Legionella pneumophila* infection [61]. Additionally, during the NLRC4-dependent response to *Anaplasma phogocytophlium*, caspase-1 and ASC KO mice have reduced Th1 responses which leads to increased susceptibility to severe infection [62]. NLRC4 and NLRP3 are both involved in response to *C. albicans* infection. While NLRP3 is activated from fungal hyphae, the mechanism of NLRC4 engagement is unknown in this context. However, mice with ASC and caspase-1 deletion display reduced Th1 and Th17 responses and were highly susceptible to fungal disease. Moreover, in the absence of ASC and caspase-1, mice infected with influenza elicited diminished CD4+ and CD8+ cell responses, as well as reduced antibody titre, in an NLRP3-dependent manner [63]. Another link for NLRP3 to the adaptive immune system is related to tumor responses. Following chemotherapy, the NLRP3 inflammasome plays an important role in priming tumor specific CD8+ T cells leading to an anti-tumor adaptive immune response [64]. Chemotherapy was not effective in NLRP3 inflammasome-deficient mice.

Several examples illustrate how NLRs act as important bridges between immune systems by secreting cytokines. NLR activity in innate immune cells is associated with the release of cytokines that stimulate adaptive immunity. However, in a reversal of roles, adaptive immune cells can act on macrophages to alter inflammasome status. For example, IL-2 activated human invariant NKT cells can activate the NLRP3 inflammasome in resting macrophages by direct contact, independent of P2X7 signalling and K^+^ efflux [65]. Additionally, direct contact from activated effector and memory CD4+ T cells can abolish NLRP1 and NLRP3 inflammasome activation in macrophages [66]. The two-directional mode of communication between adaptive and innate immune cells highlights their integrated roles in host defence.

## 2. The ATP-Dependency of NLR Activation

### 2.1. NLRs are STAND ATPases

Early studies of plant R proteins [67,68], Caenorhabditis elegans cell death gene ced-4 [69] and the mechanism of APAF-1 apoptosome assembly [70,71] provided the first indication that nucleotide binding by NLRs was likely to be a regulated process to provide control of inflammasome assembly. Furthermore, the classification of NLRs as STAND ATPases inferred that the conserved ATP binding and hydrolysis motifs likely functioned analogously to biochemically characterized non-NLR STAND proteins, which couple ATP hydrolysis to structural reorganization; thus, driving oligomeric assembly and downstream effector activation [12].

The strong sequence conservation among nucleotide binding (NB) sites shared by STAND proteins including APAF-1, plant R defence proteins and CED-4 (ARC) led to the first description of a novel protein motif, the NB-ARC domain [72]. Subsequent biochemical and functional analyses of these and other proteins with NB-ARC domain sequences suggested similarity of function in a broad group of plant and animal proteins linked to cell-death programs. For example, the elimination of biological function for multiple NB-ARC gene products was reported in the respective systems following the mutation of critical motifs responsible for nucleotide binding. Ultimately, iterative database searches of conserved and unique sequence provided rationale to categorize the NACHT domain proteins as a sister group of the NB-ARCs. As described by Koonin and Aravind in 2000 [11], seven distinct motifs allowed categorization of the NACHT domain, including the functionally important ATP/GTPase-specific P-loop (Walker A motif) and Mg^2+^ cation-binding site (Walker B motif). Subtle, yet unique, features of the NACHT domain sequence also distinguished its architecture from other STAND AAA+ NTPases; namely (1) a prevalence of small hydrophobic residues located immediately C-terminal of the Mg^2+^-coordinating Asp in the Walker B motif, (2) inclusion of another acidic residue +2 residues downstream of the Mg^2+^-coordinating Asp, and (3) a conserved pattern of polar, aromatic and hydrophobic residues in the distal C-terminal motif of the NACHT domain. Taken together, the analogous domain architectures suggest an evolutionary relationship between the NB-ARC and NACHT families [11]. Additional mechanistic similarities were presumed given that proteins in both families were favoured with a diversity of protein-protein interaction modules that would provide prolific signal transduction and scaffolding opportunities.

Comprehensive structural and biochemical analyses of STAND ATPases have outlined an activation mechanism whereby they function as tightly regulated molecular switches, with the “off” state as an ADP-bound, long-lived monomer that upon inducer sensing and ADP for ATP exchange, forms an ATP bound multimeric complex that is active, or “on” [12]. The switch mechanism relies on the specific architecture of the NACHT domain and key catalytic motifs conserved throughout the family.

ATP-bound, activated STAND proteins are known to form oligomeric assemblies by coupling ATP hydrolysis to the generation of mechanochemical work, thereby driving extensive and global conformational reorganization of protein structure and providing surface exposure of previously concealed binding sites for multimeric complex formation [73]. Nucleotide exchange and oligomerization of the resting monomer to the activated, multimeric state is associated with extensive remodeling of intramolecular and interdomain interactions.

The “STAND binary switch” postulation is supported in NLRs by studies of structural analogs APAF-1 and NLRC4 (Figure 1B) [12]. Moreover, a newly published 2019 cryo-EM structure of NLRP3-ADP bridges the divide between other STAND proteins and NLRPs and expands the understanding of mechanisms for NLR activation (Figure 1B,C) [74,75,76].

### 2.2. NLR Phylogeny

The evolutionary relationships of individual NLR family members have been profiled in detail (see [77,78,79]), and clearly delineate distinct NLR groups by their respective effector domain compositions. For example, the PYD-containing NLRP proteins cluster apart from NLRA, NLRB, NLRC and NLRX receptors with a highly significant internal branch [77], suggesting a single genomic event brought together the NACHT and PYD domains in the NLRPs. NACHT domain phylogenetic analyses suggest that NLRC4 is basal to other NLRs, that CIITA arose by gene duplication before the divergence of tetrapods from bony fishes, and that the duplication of NOD1 and NOD2 genes occurred before the divergence of birds and mammals. Within the mammalian lineage, the NLRP diversification events are less clear, with the expansion of NLRP paralogues in non-human organisms originating from diverse basal orthologues, indicating the important role for this family in innate immune defenses across various species. In humans, 9 of the 14 NLR proteins (NLRP2, 4, 5, 7, 8, 9, 11, 12, 13) are clustered together at chromosome 19, which suggests a major expansion event. NLRP6, 10 and 14 are clustered at chromosome 11, and NLRP1 and 3 are located at chromosomes 17 and 1, respectively. Sequence variations among the NLRP subfamily as they relate to ATP-binding motifs will be overviewed in upcoming sections.

### 2.3. The Importance of ATP in NLR Activation

It is now generally accepted that ATP-binding and hydrolysis are fundamental enzymatic properties of NLR proteins that provide the ability to initiate downstream signaling following inflammasome complex assembly. However, a lack of experimental data for all NLRs has obstructed holistic understanding of the biochemical mechanisms and kinetics of inflammasome activation. In fact, no study has rigorously parsed the distinct roles of nucleotide-binding and hydrolysis in the assembly of NLR inflammasomes. With a focus on the NLRP sub-family, the ATPase activity/ATP-binding has been well studied in NLRP1, NLRP3, NLRP7 and NLRP12 receptors. The reports on NLRP1, NLRP3, and NLRP7 support a critical role for the binding of ATP in oligomerization and inflammasome activation. Maharana and colleagues observed a similar mode of ADP- and ATP-binding by in silico modelling of the NACHT domain from NLRP1-14 with molecular dynamic simulations [47]. These structural models revealed a similar spatial arrangement of nucleotide-binding domain (NBD), helical domain 1 (HD1) and winged helix domain (WHD) for the entire NLRP family. Biochemical studies of the NLRP family members with P-linked ATP-Sepharose also support effective ATP-binding potential [80].

Setting these findings aside, there is potential that some NLRs could be classified as “pseudoenzymes” in the absence of biologically relevant ATP hydrolysis activity. Indeed, the NACHT domain of CED-4 was found to be constitutively occupied with ATP but did not appear to hydrolyze the nucleotide [81]. Moreover, recombinant NLRP1 did not exhibit signs of ATP hydrolysis even when incubated in the presence of MDP ligand that is known to activate the NLR [44]. Many pseudoenzymes have inherent pseudokinase, pseudoATPase or pseudoGTPase features that can provide allosteric regulation, localization and intracellular trafficking, or hubs for assembling protein complexes [82,83]. It is now apparent that pseudoenzymes constitute ~10% of proteomes and perform essential metabolic and signaling functions that can be experimentally distinguished from catalytic outputs.

ATP-binding and hydrolysis can play distinct roles in the conformational remodeling and ensuing functionality of ATPases (e.g., 26S proteasome, myosin, EccC type VII secretion system). Given disparities in the precise NACHT sequence identified within several NLRPs, it is justifiable to predict that distinctions in the kinetics and effect of ATP hydrolysis exist for the various family members. While the ATP-coordinating regions of the NBD are practically invariant (i.e., Walker A motif), there is significant variability within motifs that are thought to participate in hydrolysis and the subsequent conformational rearrangement that is required for self-oligomerization [78,84,85]. It is also likely that activation kinetics will differ since separate conformational changes would be transmitted globally to other domains first with ADP to ATP exchange in the active site, and subsequently when ATP is hydrolyzed and/or ADP released. Finally, it is expected that unique enzymatic properties of the NACHT domain in different NLRs will drive distinctions in nucleotide interaction, activation state and in the regulation of inflammasome assembly and immune signaling.

## 3. ATP-Dependency for the Assembly and Activation of Selected NLR Inflammasomes

### 3.1. NLRC4

NLRC4 (formerly known as IPAF) can activate pro-caspase-1 without recruiting ASC [4,38,86]. Absent a PYD domain, this is achieved by direct recruitment via a CARD domain present in NLRC4. Despite this, the co-expression of ASC in experimental model systems appears to greatly enhance the efficacy of NLRC4 inflammasome assembly. Nonetheless, pyroptosis mediated by NLRC4 has been observed to occur independent of ASC. NLRC4 is broadly expressed, but it displays enhanced expression in blood and lymphoid tissues [48,49,50]. This includes in ranked descending order: spleen, lymph nodes, appendix, and bone marrow. With respect to immune cell expression, NLRC4 is enhanced in monocytes, dendritic cells and granulocytes.

Bacterial flagellin is the canonical activating stimulus for NLRC4. This PAMP is detected by an NLR apoptosis-inhibitory proteins (NAIPs) that bind the cognate ligand and oligomerize with NLRC4 to form an active inflammasome [87,88]. NAIPs are cytosolic NLRB family members with baculovirus IAP repeat (BIR), NACHT, and LRR domains [10]. Mice express several (up to 7) NAIPs while a single NAIP ortholog (NLRB) is found in humans. Different NAIP paralogs dictate the specificity of the NLRC4 inflammasome for distinct bacterial ligands. Recognition of T3SS rod protein (NAIP2), T3SS needle protein (NAIP1), and flagellin (NAIP5, NAIP6) by the respective NAIPs enables binding to murine NLRC4 [87,88]. Legionella infection was also shown to drive a NAIP5/flagellin-dependent inflammasome response [89]. However, NLRC4 activation following Samonella infection was only partially dependent on NAIP5. Additional studies revealed the T3SS rod protein, PrgJ, found in Salmonella but not Legionella, to act as a PAMP with activation of NLRC4 through NAIP2 [87,90].

Ligand sensing by NAIP proteins is not conferred by the LRR as previously assumed but by specific regions of the central NACHT domain. Specifically, HD1, WHD and helical domain 2 (HD2) motifs were deemed necessary and sufficient to confer ligand specificity [91]. NAIP binding to NLRC4 via the NACHT domain relieves LRR-mediated autoinhibition. A sequential process then occurs whereby NLRC4 monomers are recruited into an active conformation. Eventually a 10–12-mer wheel of NLRC4 proteins is formed that acts as the structural platform for pro-caspase-1 recruitment, forming an extended filament [15,92,93,94]. More recently, Li and colleagues employed Cryo-EM and structural modeling to further refine the NLRC4 inflammasome filaments that are mediated through CARD-CARD interactions. They described a structural mechanism whereby ASC and NLRC4 adopt similar molecular interfaces and assembly patterns to nucleate pro-caspase-1 via homotypic CARD-CARD interactions [95].

Lu and colleagues first described the nucleotide-binding properties of NLRC4 by application of recombinant GST-fusion proteins in a radionucleotide-binding competition assay [96]. ATP was determined to be the preferred ligand with an estimated *K*_d_ in the nM range, while CTP and GTP were unable to compete with bound [^33^P]-ATP. A mutation made in the P-loop of the Walker A motif nearly abrogated ATP binding. The study further resolved that a functional NACHT domain was required for self-assembly and pro-caspase-1 activation. Notwithstanding the importance of homotypic CARD-CARD interactions in promoting inflammasome assembly, the need for a functional NACHT domain is apparent since this region provides one of two important symmetries for the interaction of full-length NLRC4 filaments [95].

NLRC4, like other NLRs, is involved in a variety of autoinflammatory syndromes via hyperactivating mutations that result in excessive IL-1 family cytokine production. A NLRC4 missense mutation (i.e., T337S) was discovered in a patient with recurrent macrophage activation syndrome (MAS) [97]. An independent group identified a similar NLRC4 missense mutation (i.e., V341A) that was associated with infantile enterocolitis, periodic fever, and fatal or near-fatal autoinflammatory syndromes in a family [98]. The mutations were associated with NLRC4 gain-of-function phenotypes, macrophage cell death and constitutive caspase-1 cleavage in concert with enhanced IL-1β and Il-18 production. The two mutations were localized to the HD1 motif of the NACHT domain, and mechanistic insights from both groups, based largely on the crystal structure of NLRC4 published by Hu and colleagues [15], suggest the residues are critical for stabilizing ADP/ATP binding in the NACHT domain and are thought to cause hyperactivation of NLRC4 by abrogating autoinhibition by the LRR domain. Overall, these mutations and other mutations associated with MAS induce functional impacts that are very similar to those observed for cryopyrin-associated periodic syndrome (CAPS) associated with mutations in the NACHT domain of NLRP3 [75].

### 3.2. NLRP1

NLRP1 was the first inflammasome platform to be defined and is structurally similar to the well characterized apoptosome apoptotic protease-activating factor 1 (APAF-1), that is involved in apoptosis [30]. The activation mechanism and biological impact of NLRP1 inflammasomes (and its murine NLRP1b homolog) are more fully established given a wealth of recent research activities [99,100]. NLRP1 displays low tissue specificity as it is found in significant amounts in a wide variety of tissues. However, this profile is consistent with the enhanced expression in lymphoid tissue seen with many NLRs and is in line with NLRP1′s role in inflammation. With respect to immune cells, NLRP1 is expressed mostly in neutrophils and basophils, but it is also present in a variety of other cell types, including monocytes, T-cells, and B-cells [48,49,50]. This is unique in the NLRs, few of which have any expression in T and B cells. Humans only have a single copy of NLRP1, whereas rodents have multiple paralogues (NLRP1a, b, c).

A functional degradation mechanism is proposed to account for the activation and assembly of NLRP1 inflammasomes. In this process, N-terminal proteolysis of NLRP1 within the FIIND domain elicits inflammasome activation and induction of an inflammatory response. The proteolytic event liberates a NLRP1 C-terminal fragment that is then able to recruit and activate caspase-1 via homotypic CARD-CARD interactions [99,100]. The microbial cell wall component muramyl dipeptide (MDP) is the only known ligand for NLRP1, although anthrax lethal toxin, *Plasmodium vivax* malarial infection, and *Toxoplasma gondii* are associated with PAMPs that provide rapid activation of NLRP1 inflammasome signaling [99,100]. Dysregulation of the NLRP1 inflammasome has been implicated in a variety of autoimmune diseases [9]. Polymorphisms in NLRP1 to be associated with increased susceptibility to rheumatoid arthritis (RA), multiple sclerosis (MS), and systemic lupus erythematous (SLE) [60]. IL-1β secretion is host-protective in *Toxoplasma gondii* and *Plasmodium vivax* infection; this suggests deleterious SNPs reduce NLRP1 activity. In contrast, elevated IL-1β levels are disease causing in RA, MS, and SLE. This implicates this set of NLRP1-associated mutations as causing inflammasome hyperactivity. In both cases, the exact disease-causing mechanisms of the mutations are not known.

There are inconsistencies in the literature regarding the role of nucleotides as cofactors for NLRP1 inflammasome assembly. In an early study, a reconstituted NLRP1 inflammasome was manufactured with Sf9 insect cells and a baculovirus expression system and then used to profile the nucleotide dependence of NLRP1 activation [35]. NLRP1-dependent activation of pro-caspase-1 required ATP (and Mg^2+^ as a cofactor) while ADP was unable to elicit activation. Nonhydrolyzable analogs of ATP and GTP failed to support NLRP1 activation, indicating NTP hydrolysis may play an important mechanistic role. All triphosphates (e.g., ATP, GTP, CTP, TTP, and UTP) were able to support NLRP1-dependent pro-caspase-1 activation; however, ATP was judged to be the most efficacious. This result was supported in vitro by fluorescence polarization and scintillation proximity assays wherein displacement of fluorescein-12-ATP or [γ^35^S]-ATP, respectively, from recombinant GST-NLRP1 was most effective with ATP [101]. The ATP-binding of recombinant GST-NLRP1 was also significantly diminished by mutation of the Walker A motif. Ultimately, Faustin and colleagues concluded that activation of the reconstituted NLRP1 inflammasome occurred in a two-step mechanism whereby binding of the muramyl dipeptide (MDP) ligand rendered NLRP1 competent to bind ATP with subsequent inflammasome assembly via oligomerization of NACHT domains [35]. Mutational ablation of ATP binding prevented oligomerization of NLRP1 monomers into a multimeric complex.

Additional examinations of NLRP1 structure by SAXS analysis found a soluble fragment of the protein to be constitutively bound with ATP and to have negligible enzymatic activity (i.e., ATP hydrolysis was not observed) [44]. In this case, provision of MDP ligand did not promote self-oligomerization of the NACHT-LRR fragment suggesting that MDP may bind to regions outside the NACHT and LRR domains. In contrast, Liao and colleagues found a silencing mutation of the Walker A motif, that would prevent ATP binding, to enhance murine NLRP1b inflammasome activity [102], and these authors concluded that the assembly of a functional NLRP1b inflammasome was not ATP-dependent. When compared against its wild-type control, the Walker A mutant of NLRP1b was judged to be constitutively active since an increase in Il-1β secretion was observed in cell assays. This result contrasted with the abolition of activity observed when a similar silencing mutation was introduced into the Walker A motif of NLRP3. While the ATP-binding properties of NLRP1 to immobilized ATP-Sepharose are similar to other human NLRP family members [80], the NACHT domain of murine NLRP1b may have different requirements for ATP than that of human NLRP1. Ultimately, future clarification will be required since the studies to date have employed both murine and human homologues as well as different model systems for the assessment of inflammasome signaling (i.e., cell-based and recombinant proteins).

### 3.3. NLRP2

NLRP2 can form an inflammasome with ASC and caspase-1 to provide maturation of pro-inflamammatory IL-1β and IL-18 [103,104]. Notably, Minkiewicz et al. (2013) showed that NLRP2 was activated in astrocytes by ATP via the P2X7 receptor and the pannexin 1 channel, similar to mechanisms reported for NLRP3. NLRP2 activation of caspase-1 resulted in IL-β and IL-18 secretion that was reversible in the presence of a pannexin 1 inhibitor. Overall, these data suggest astrocytic NLRP2 is likely an important component of inflammatory response in the central nervous system (CNS) [103]. Another study further solidified the role of NLRP2 in CNS inflammation using a mouse model [104]. NLRP2 was significantly upregulated in dorsal root ganglion as were, although to a lesser extent, NLRP3 and NLRP1. Importantly, knockdown of NLRP2 by siRNA abolished inflammatory responses and pain hypersensitivity from complete Freund’s adjuvant or ceramide treatment [101]. Conversely, additional investigations have shown NLRP2 to act as an inhibitor of NF-κB signaling in various cell types [105,106,107]. NLRP2 interacts with IKK to suppress NF-κB activation [105,107]. NLRP2 also reduces tumor necrosis factor (TNF)α-induced phosphorylation of the NF-κB p65 subunit [106]. In the presence of TNFα and IFNγ, NLRP2 provides enhanced major histocompatibility complex expression in trophoblasts to promote a more robust immune response. NLRP2 was associated with attenuated human leukocyte antigen (HLA)-C expression in fetal extravillous trophoblasts. HLA-C has a unique role in pregnancy since it is the only polymorphic major histocompatibility complex molecule that can elicit an allogeneic response by maternal T cells at the maternal–fetal interface. Tilburgs et al. (2017) also showcased a role for NLRP2 in bridging the innate and adaptive immune systems in trophoblasts [106]. Disparate findings for the activating and inhibitory properties of NLRP2 may be partially explained by unique functionalities for the inflammasome in different cell types.

NLRP2 has enhanced expression in testis and ovaries, and several studies have demonstrated an importance for NLRP2 in embryonic development [108,109,110,111,112]. For example, maternal depletion of *Nlrp2* in zygotes in mice led to early embryonic arrest [108]. In contrast, Kuchmiy and colleagues showed that NLRP2-deficient mice were born with expected Mendelian ratios; however, these authors noted declining reproductive rates with progressing age of female *Nlrp2*-deficient mice [109]. This suggests that NLRP2 variants with reduced activity may contribute to maternal age-associated fertility loss in humans. These studies also reported no linkage between NLRP2 and the innate and adaptive immune systems since no differences in CD4+ T cell activation were observed. NLRP2 is expressed highly and selectively in oocytes. Following fertilization, *Nlrp2* expression rapidly decreases [109]. This is in keeping with its role in embryo implantation. It is worth noting that the above embryonic roles of NLRP2 are not necessarily linked to inflammasome assembly and activation. It is possible NLRP2 acts outside of an inflammatory context to impact on embyogenesis [110]. Interestingly, NLRP2 and NLRP7 are paralogues that arose from a whole genome duplication event. Both proteins have similar roles in early embryo tolerance and development.

There is a paucity of studies that examine the importance of the NACHT domain in NLRP2 inflammasome assembly and downstream signaling. While structural modeling supports the ability of NLRP2 to bind ATP [47], NLRP2 was unique amongst the NLRPs in displaying low elution efficiency from immobilized ATP-Sepharose resin. This could indicate lower comparative efficacy in ATP binding [80]. Of note, evidence suggests that the NLRP2 inflammasome also contains CARD8, a caspase recruitment domain-containing protein. CARD8 possesses a FIIND domain that cooperates with the NLR protein to assemble an inflammasome complex with pro-caspase-1. The interaction of NLRP2 with CARD8 was shown to require the NACHT domain and was internally suppressed by the LRR and PYD domains in the absence of inflammasome activation [113]. Single nucleotide polymorphisms within the NACHT domain were proposed to contribute to the amplification of NLRP2-dependent inflammatory responses due to a reduction of inhibitory signals on NF-κB [114]. A polymorphic change that gives rise to a non-functional NLRP2 protein (i.e., Ile352Ser) was located within an arginine finger motif that is predicted to affect the efficiency of ATP hydrolysis.

### 3.4. NLRP3

NLRP3 is expressed predominately in bone marrow and basophils, with more modest expression in dendritic cells, monocytes, gallbladder, lymph node, appendix, and spleen [48,49,50]. This broad expression profile is in line with its ubiquitous role in inflammation. Indeed, the aberrant activation of the NLRP3 inflammasome has been shown to drive numerous chronic inflammatory diseases including atherosclerosis, gout, rheumatoid arthritis, ischemic stroke, and many more [3]. NLRP3 is the most widely studied protein in the NLRP family, and serves as a prototypical model for canonical inflammasome activation [19,21,85,115]. Signal One, the priming step, involves PAMP or DAMP recognition (e.g., LPS by TLR4) which increases the transcriptional expression of NLRP3, IL-1β and IL-18. In the canonical pathway, this is accomplished by a TLR4/Myd88/TRIF/MAPK/NF-κB signaling pathway. Additionally, in the case of NLRP3, dephosphorylation and deubiquitinylation are required during the priming step. The Second Signal activation step involves ATP-dependent oligomerization of the NLR with ASC via homotypic PYD-PYD interactions and with pro-caspase-1 via CARD interactions. Pro-caspase-1 undergoes autocleavage and dimerization into its active form, which cleaves and matures IL-1β and IL-18. GSDMD is also cleaved by the inflammasome, the resultant N-terminal fragments form membrane pores which enables cytokine release, ion depolarization and membrane rupture, completing pyroptosis [6,7]. Activating PAMP and DAMP signals for NLRP3 are famously diverse and include: K^+^ efflux, Cl^-^ efflux, Ca^2+^ influx, mtDNA, alum, reactive oxygen species (ROS), implanted biomaterials or medical devices, asbestos, silica, calcium oxalate, and uric acid crystals, as well as a variety of metabolic components including cholesterol crystals and succinate accumulation [4,38,56,115,116]. Moreover, the NLRP3 inflammasome is thought to be a major pathophysiologic contributor to the cytokine storm observed in the clinical course of patients with COVID 19 [117,118,119]. There also exists a mechanism for non-canonical inflammasome activation involving caspase 4 and 5 in human, and caspase-11 (homologue) in mice [9]. Intriguingly, activation of this non-canonical pathway also results in the downstream activation of the canonical NLRP3 inflammasome via K^+^ efflux.

First reported by Duncan and colleagues in 2007, a functional NACHT domain is required for biological activity of the NLRP3 inflammasome [41]. The ATP-binding properties of recombinant NLRP3 protein were revealed by association with immobilized ATP-agarose resin. NLRP3 also exhibited ATPase activity as release of ^32^P-orthophosphate was observed following incubation of NLRP3 protein with [γ^32^P]-ATP. Mutation of the Walker A motif abrogated in vitro ATP-binding as well as inflammasome-mediated IL-1β secretion from THP-1 cells. Functional ATP-binding by the NACHT domain was also required for the effective assembly of an NLRP3 inflammasome complex.

More nuanced involvement of the NACHT domain in NLRP3 inflammasome biology has been revealed recently. Upon activation, NLRP3 assembles in the perinuclear space and appears to associate with mitochondria. Nucleation however, likely begins in the dispersed trans-Golgi network, where basic residues between the PYD and NACHT domains bind phosphatidylinositol-4-phosphate (PtdIns4P) present on the surface of Golgi bodies [120]. In addition, phosphorylation of NLRP3 within the NACHT domain at Ser295 has important ramifications on inflammasome activity [85]. First, Ser295 phosphorylation was detected following NLRP3 self-oligomerization, and it was suggested that this phosphorylation event could release NLRP3 from mitochondria-associated ER membranes, allowing for the inflammasome assembly [121]. Second, Ser295 phosphorylation of NLRP3 was linked to the attenuation of ATPase activity, the rapid inhibition of inflammasome signaling, and decreased inflammatory responses [122,123]. Molecular modeling has revealed putative conformational changes in protein structure that may accompany Ser295 phosphorylation and lead to changes in ATP-binding or hydrolysis efficacy [85].

The mitotic Ser/Thr NIMA-related kinase (NEK7) has proven to be an important modulator of NLRP3 inflammasome activation [76,124,125,126]. Interestingly, NEK7 was required for NLRP3-activating mutations within the NACHT (i.e., R258W, mouse numbering) to provide caspase-1 activation and IL-1β maturation [76,125]. Cryo-EM and molecular modeling of a NEK7-NLRP3 complex revealed molecular interactions between the C-terminal lobe of NEK7 and NLRP3 that were dependent on two distinct interfaces [76]. One half of the C-lobe of NEK7 interacted with the LRR domain and the other half associated with the NACHT. Site-directed mutagenesis further resolved the importance of the NBD and HD2 regions of NLRP3 for the binding of the kinase. Incubation of the inactive NLRP3-NEK7 heterodimer with ATP was unable to induce in vitro oligomerization. Interestingly, the NBD of NLRP3 appears to lose molecular contacts with NEK7 in a model of active NLRP3. So, NEK7 binding of NLRP3 may “licence” the protein for activation but is still insufficient for inflammasome assembly without additional allosteric triggers acting to convert the NACHT domain from inactive to an active conformation [76]. These newly-emerged complexities of NEK7-dependent control of NLRP3 inflammasome formation will require further investigation in order to fully understand the inherent role of ATP binding and hydrolysis.

There are several hereditary diseases with NLRP3 inflammasome involvement that are categorized as cryopyrin-associated periodic fever syndromes (CAPS) [127]; these include familial cold auto-inflammatory syndrome (FCAS), Muckle-Wells syndromes (MWS), and neonatal-onset multisystem inflammatory disorder (NOMID). There are over 200 documented CAPS-associated mutations for NLRP3 in the INFEVERS database [128], and many of these mutations are concentrated in the exon that encodes the NACHT domain. Some of these protein variants, when over-expressed self-assemble and activate in the absence of stimuli, supporting a gain-of-function disease model [127,129]. Indeed, CAPS-associated mutations of NLRP3 are proposed to alter the conformation of the protein to provide a reduced activation threshold and thus an inflammasome complex that is capable of responding to reduced amounts of activating ligands [129]. Duncan and colleagues provided proof-of-principle that CAPS-associated variants of NLRP3 still required nucleotide-binding properties for activation [41]. IL-1β maturation was augmented when a FCAS/MWS-associated mutation (i.e., Arg260Trp) was introduced into NLRP3. Mutation of the Walker A motif to prevent ATP-binding ablated the IL-1β secretion of both wild-type and Arg260Trp NLRP3 inflammasomes. An additional study revealed that disrupting the ATP-binding efficacy of NLRP3 with mutation of the Walker A motif could inactivate a constitutively-active variant of the NLRP3 inflammasome that possessed an Arg258Trp CAPS-associated mutation (mouse numbering) within the NACHT domain [102]. Ultimately, these CAPS-associated mutations may alter the biological action of the NLRP3 inflammasome by inhibiting ATPase catalytic activity, influencing the ADP/ATP exchange or altering the ATP substrate binding affinity. Even with significant advances in understanding, the precise role of ATP hydrolysis in NLRP3 inflammasome assembly or disassembly still remains to be fully characterized at a biochemical level.

### 3.5. NLRP6

NLRP6 is a multifaceted NLR that regulates inflammasome-dependent and inflammasome-independent host defense responses. NLRP6 is expressed in the lung, liver, kidney, as well as the small and large intestines [48,49,50]. Expression is largely specific to intestinal epithelial cells, including the small intestine, duodenum, and colon. NLRP6 is also expressed, although to a lesser degree, in granulocytes. Overall, this reflects an important role for NLRP6 in maintaining gut homeostasis [130,131,132]. In this regard, NLRP6 has been implicated in regulating autophagy and mucus granule exocytosis in goblet cells, orchestration of antimicrobial peptide secretion, intestinal microbial colonization, intestinal tumorigenesis, epithelial regeneration, viral RNA recognition of (+) ssRNA and encephalomyocarditis virus [133], and regulation of inflammatory signaling in myeloid cells. While the precise mechanisms for initiation of these diverse roles remains to be clarified, the consensus view is that each function is cell type specific. In line with the various roles NLRP6 has in maintaining intestinal homeostasis, several gut-inflammatory diseases have been described for NLRP6 mutants.

NLRP6 deficiency leads to gut dysbiosis and the development of an inflammatory microbiome profile. Interestingly, the harmful microbiome can be transferred from *Nlrp6*^−/−^ to wild-type mice where the dysbiosis persists. This can be explained by the negative regulation of NLRP6 by microbial metabolites spermine and histamine, discovered in an unbiased metabolomic study [134]. Subsequent deficiency in downstream IL-18 and antimicrobial peptides further enables aberrant microbial colonization. In addition, NLRP6 was shown to play a host protective role in chemically-induced colitis by enhancing activity of infiltrating intestinal Ly6C^hi^ inflammatory monocytes and neutrophils. Adoptive transfer of Ly6C^hi^ monocytes from wildtype mice to *Nlrp6* deficient mice resulted in protection from colitis, a reduction in barrier permeability, lower bacterial translocation, and increased survival [135]. In contrast, non-canonical NLRP6 signaling acts to inhibit NF-kB and MAPK pathways to reduce inflammation in monocytes. Accordingly, *Nlrp6*^−/−^ mice display elevated levels of inflammatory cytokines such as IL-6 and TNFα, greater resistance to *Listeria monocytogenes* and *Salmonella typhimurium*, as well as abrogated mucus secretion from goblet cells [136,137].

Two reports have been produced on the structure of NLRP6, and the two models of inflammasome assembly are very different from one another. One report describes assembly of a cylindrical polysome whereby oligomerization of NLRP6 via its PYD regions forms a filament that permits further nucleation with ASC as the supramolecular platform for recruitment of pro-caspase-1 via CARD-CARD interactions [138]. The other report describes a novel mode of oligomerization involving NLRP6 homodimers as a linear repeating unit that integrate with ASC to form higher order filamentous structures [139]. A two-step mechanism for NLRP6 oligomerization was proposed in this latter study, with ligands such as LPS binding directly to the monomer to initiate NLRP6 dimerization followed by ATP-dependent oligomerization of the NLRP6 dimers to provide a linear platform for the association of adaptor protein ASC.

Using cryo-EM and X-ray crystallography, Shen and colleagues found the homotypic PYD-PYD interactions of the NLRP6 pyrin domain (i.e., NLRP6^PYD^) assembled to form a hollow cylindrical filament with an outer diameter of 8 nm [138]. This assembly was enhanced nearly 10-fold for an NLRP6 construct that also contained the NACHT domain (NLRP6^PYD-NBD^), as opposed to NLRP6^PYD^ alone, and supports a role for ATP-binding and/or hydrolysis in mediating homotypic PYD-PYD assembly of the NLRP6 inflammasome. This finding was similar in nature to that described for the assembly of NLRP3^PYD^ which was dependent on the presence of the NACHT domain as well [140]. Over-expression of full length NLRP6 yielded filamentous structures with a diameter of approximately 30 nm characterized by a high-density core originating from PYD assemblies with the NACHT and LRR domains on the exterior. The NACHT domains then associate in the outer ring to synergize with the PYD core to form an interface for ASC. This model differs somewhat from the proposed NLRC4 inflammasome structures [15,92,93,94]. For NLRC4, the NLR protein oligomerizes to form a wheel with a CARD filament forming the central core that acts as the site of unidirectional filament nucleation to complete the inflammasome superstructure. In contrast, the Shen et al. suggest the NLRP6 inflammasome contains a dense PYD core with the NACHT and LRR domains projecting outwards in a less-ordered arrangement. An NLR-wheel-head model of nucleation was described for NLRC4 [93,94] and suggested for NLRP3 inflammasomes [76]. NLRP3 and NLRC4 oligomerize and remain in a bundle at the head of the filament but are not a part of the composition along the length of the filament, unlike that suggested for the NLRP6 inflammasome where the NLR is present along the entire filament length. Notably, active NLRP6 inflammasomes were not reconstituted, rather only NLRP6 was over-expressed and allowed to self-assemble. So, it is possible that the activating ligand or co-expression of adaptor protein ASC and pro-caspase-1 could alter the NLRP6 filament structure to one that resembled the NLRC4 superstructure.

In contrast, Leng and colleagues [139] uniquely suggest that NLRP6 can form pre-inflammasome structures. Lipopolysaccharides (LPS) bound NLRP6 directly with high-affinity and could induce NLR protein homodimerization. The additional exposure of NLRP6 homodimers to ATP produced oligomers in a concentration-dependent manner. Each NLRP6 homodimer is formed by anti-parallel interactions between two LRRs, forming the major interface for dimerization. Two homodimers interact via NACHT-NACHT interactions to form higher order oligomers with a 120° rotation observed between dimer units of a NLRP6 hexamer. These platforms were suggested to serve as pre-inflammasome structures, available and able to accommodate ASC and pro-caspase-1. The interactions between LRR domains may relieve auto-inhibition, akin to NAIP binding NLRC4. Notably, ATP alone was insufficient to induce oligomerization, rather LPS and ATP were required for higher order assembly. This suggests a two-step model of activation, as was described for NLRP1 [35] and NLRP3 [76]. Moreover, NLRP6 and ASC co-localize to the perinuclear space following LPS treatment. This was similar to the location of NLRP1 and NLRP3 assembly.

Overall, the two assembly models for NLRP6 are quite different. In Leng et al., the filament assembly was driven by NLRP6 homodimers that repeat in a linear fashion [139], whereas Shen et al. observed a continuous, unidirectional, rotating assembly of based on PYD-PYD interactions [138]. A notable difference exists between the two reports in the conditions used to promote NLRP6 inflammasome assembly. For example, Shen and colleagues relied on auto-assembly of purified NLRP6 proteins and did not use ATP or LPS to stimulate nucleation of NLRP6 oligomerization. Ultimately, further structural investigations will be required before any consensus mechanism of assembly and structure can be confidently described for NLRP6.

### 3.6. NLRP7

The NLRP7 inflammasome is a NLRP7-ASC-caspase-1 multimeric complex that provides maturation of IL-1β and IL-18 in a canonical fashion. This included robust NLRP7-dependent responses to PAMPs liberated by *Mycobacterium bovis* Beijing, Mycoplasma spp., heat-killed *Acholeplasma laidlawii*, gram-positive *Staphylococcus aureus* and gram-negative *Legionella pneumophilia* [141,142]. A host-protective role was also demonstrated for NLRP7-deficient murine bone marrow derived macrophages (BMDMs) which had increased exposure to PAMPs during *S. aureus* and *L. monocytogenes* infection [141].

Notably, NLRP7 was shown to respond to microbial lipopeptides in the absence of any non-canonical inflammasome activity [141], and inflammasome formation was dependent on ATPase activity [43]. Specifically, the Walker A motif was required for nucleotide binding and hydrolysis. Mutation of this region of the NACHT domain hindered the ability to form high molecular weight assemblies of NLRP7 in an in vitro inflammasome reconstitution assay, suggesting NLRP7 oligomerization was ATP dependent.

Various structural examinations of NLRP7 have been reported in the literature. The three-dimensional structure of the isolated NLRP7 pyrin domain was obtained by NMR spectroscopy [143]. Furthermore, the intermolecular interfaces for multimeric NLRP7 complexes were explored using a combination of yeast two-hybrid and co-immunoprecipitation with subsequent application to a homology model of the NLRP7 inflammasome superstructure generated with the APAF1 backbone [144]. When modelling of the activated and non-activated NLRP7 protein was completed, several important structural changes were proposed. Notably, these reveal important contributions of the NACHT domain and adjacent regions (i.e., a NACHT-associated domain, NAD) of the oligomeric assembly of activated NLRP7. In the inactive form of NLRP7, the NAD domain was buried and unavailable for interaction; however, this region became accessible in the activated form. Additionally, molecular movement of the PYD and NACHT domain upon activation revealed other regions of NLRP7 that had been sequestered in the inactive topology. The homology model supports the interaction of the NAD and a small region of the LRR domain of one NLRP7 molecule with the NACHT domain of a second molecule. The areas localized to the conserved Walker A and Walker B motifs within the NACHT domain appeared to be most relevant to the putative oligomerization interface.

### 3.7. NLRP9

NLRP9 is highly expressed in primary human IEC organoids but not transformed intestinal epithelial cell lines [48,49,50,145]. The mouse ortholog NLRP9b provides protection against rotavirus infection in intestinal epithelial cells where it forms a NLRP9b-ASC-caspase-1 inflammasome to produce IL-1β and IL-18, and GSDMD to induce pyroptosis [145]. Zhu et al. (2017) also showed that NLRP9b was activated following DNA helicase DHX9 recognition of rotavirus dsRNA. This finding was revealed using rotavirus-infected mouse models (NLRP9b) and infected HEK293T cells that were co-transfected with NLRP9 and ASC. DHX9 co-precipitated with NLRP9b, suggesting a direct interaction to promote assembly [145]. Here, GSDMD-mediated pyroptosis was shown to be crucial for host defence, likely since pyroptosis prevents extended viral replication by inducing premature host cell death. Although the role of the NACHT domain in regulating the assembly of NLRP9 inflammasome has not been examined, a few reports suggest a functional importance for ATP. NLRP9 could be recovered from immobilized ATP-Sepharose resin [80], which suggests effective ATP-binding. Moreover, 3D modeling of the NACHT domain suggests stable coordination of ADP and ATP in the binding pocket of NLRP9, although comparatively fewer H-bonds were identified with molecular dynamic simulations using a manually-docked complex [47]. Finally, structures of the PYD from the human NLRP9 gene reveal a novel orientation for the domain [146,147] which may reflect differences in oligomerization and ATP-dependent inflammasome formation.

### 3.8. NLRP12

NLRP12 was one of the first NLR proteins described to activate pro-caspase-1 synergistically with ASC to generate pro-inflammatory signals [148], which occurs via TLR4 signalling and formation of an ASC-caspase-1 inflammasome in response to *Yersinia pestis*, the causative agent of bubonic plague [149]. NLRP12 has enhanced expression in immune cells such as granulocytes, dendritic cells and macrophages [48,49,50]. It is associated with the negative regulation of inflammation, and its expression is down-regulated in response to PAMPs and DAMPs [150]. Indeed, multiple reports highlight the importance of NLRP12 in suppressing colon inflammation and tumorigenesis in experimental models of colitis by negatively regulating NF-κB and MAPK pathways. Canonical and non-canonical NLRP12 inflammasome activity has also been shown to negatively regulate NF-κB signaling in T cells as well [150]. *Nlrp12*^−/−^ mice had increased peripheral CD4+ and CD8+ T cells which indicates a role for NLRP12 in the regulation of mature T cell development and adaptive immunity.

The NLRP12-mediated suppression of NF-κB was shown to be dependent on ATPase activity and specifically required intact Walker A and B motifs for biological effectiveness [42]. Moreover, these motifs were required for NLRP12 to self-oligomerize in immunoblot assays using transfected HEK293T cells. The stable expression of a Walker A/B mutant in THP-1 monocytes results in increased production of pro-inflammatory cytokines and chemokines to an extent comparable to that in cells in which NLRP12 was silenced via shRNA exposure [42]. An Arg352Cys missense mutation [151] and an Arg284 nonsense mutation [152] were identified in patients with periodic fever syndromes by direct sequencing; both of these mutations were located within the NACHT domain of NLRP12. The former, while not altering the inhibitory impact of NLRP12 on NF-κB activation, was associated with increased ASC speck formation and caspase-1 processing of pro-inflammatory cytokines. The latter NLRP12 variant was associated with decreased inhibitory action on NF-κB signaling. Collectively, the results of these studies suggest the importance of the NACHT domain in regulating the anti-inflammatory activity of NLRP12.

## 4. A Molecular Description of the NACHT Domain: Key Functional Motifs

**Figure 2:**
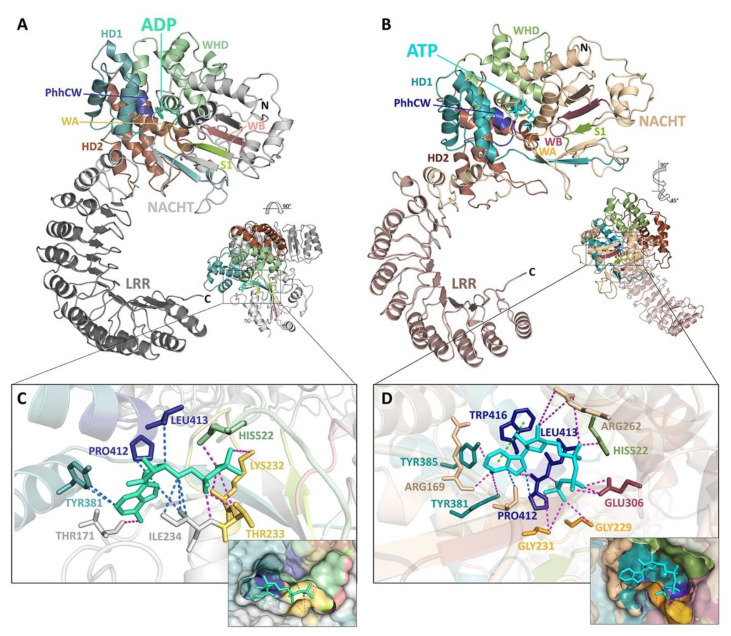
Molecular dynamic simulations of NLRP3 support distinctions in nucleotide-binding domain structure with occupancy of ATP or ADP ligands. (**A**) Ribbon diagrams in two orientations of the NLRP3-ADP structure (PDB: 6NPY). Domains are colour-coded and labelled following visual rendering with PyMOL v2.4 (pymol.org). The bound ADP ligand is shown in stick rendering. (**B**) Ribbon diagrams in two orientations of a representative ATP-bound structure provided with a 10 ns molecular dynamics simulation run with ATP occupancy of the NLRP3 structure (provided by PDB: 6NPY). Domains are colour-coded and labelled, and the bound ATP is shown with stick rendering. The detailed interactions for specific main chain and side chain residues are shown for NLRP3 and ADP (**C**) determined using the 6NPY structure, or NLRP3 and ATP (**D**) determined by molecular dynamic simulation. Insets: magnified ligand pocket surface views of the nucleotide-binding domain showing the motifs involved in coordination of the ADP (C) or ATP (D) ligands.

Members of the AAA+ superfamily feature a conserved P-loop or Rossman fold that adopts a three-layered α-β sandwich configuration [78]. A core, parallel β-sheet arranged in a 51432 topology is bounded by two α-helices (Figure 1B, Figure 2A,B). Key motifs within the Rossman fold mediate nucleotide binding and hydrolysis and initiate both short- and long-range conformational changes in the scaffold protein [78,153].

As depicted in Figure 1C, NLRPs possess a globular NTPase domain known as NACHT, which in turn is composed of a core NBD. NLRs, as STAND ATPase protein family members, are differentiated from other P-loop NTPases by the requisite Walker A (WA), Walker B (WB) and Sensor 1 (S1) motifs associated with the N-terminal helix and the core strand-4. NLRs are also differentiated by the presence of a C-terminal helical bundle fused to the NBD containing the HD1 (including the PhhCW motif situated between the two distal helices), the WHD, and the HD2 [13,78,84]. This extended NBD is obligate for NLR enzymatic activity and provides ATP binding and hydrolysis properties. This is in accordance with NLR membership in the STAND family, a part of the AAA+ ATPase superfamily, which typically rely on ATP hydrolysis to execute function [12,13].

Residues within the ATP-binding WA motif form a flexible loop between strand 1 and helix 1, and provide the necessary electrostatics to coordinate the α- and β phosphates of ATP and contribute electron withdrawing groups to facilitate the H_2_O-Mg^2+^ nucleophilic attack coordinated by residues present in the ATP-hydrolysing WB motif. The WA motif is defined by a characteristic consensus sequence (i.e., GxxxxGK(S/T), where x indicates any amino acid residue and alternative residues are shown in brackets [78]). A recently published 3.8 Å cryo-EM structure of NLRP3 confirms in an NLRP member that the conserved Lys residue (NLRP3: Lys232) within the WA motif does provide direct interaction with the γ-phosphate of the nucleotide (Figure 1A, Figure 2C,D), as does the succeeding Thr (providing additional stabilization to the β phosphate) [76]. Proell and colleagues have previously suggested with evolutionary analyses that the presence of a Thr or Ser residue in the GK(S/T) sequence of WA could be used to further subdivide the NLR family into two groups [78]. NLRP1, 5 and 12, along with the primordial NLRA, NLRB, NLRC and NLRX subfamilies, contain a Ser residue while the remaining NLRPs contain a Thr. Since both groupings contain members with demonstrated ATPase activity, the similar physical properties of both hydroxylic residues likely serve similar roles in ATP binding.

The Walker B (WB) motif is crucial for ATPase activity, with a consensus sequence of hhhh(D/E) (where h represents a hydrophobic amino acid). The acidic Asp or Glu residues coordinate the Mg^2+^ cation, which is in turn coordinated to the β- and γ-moieties of the substrate nucleotide, and activate a water molecule for hydrolysis, respectively. Innovative ‘substrate-trap’ mutants elucidated the dispensable role of the WB motif in ATP binding. Indeed, the mutation of the Glu residue blocked nucleotide hydrolysis but not binding [154]. In the NLRP family, the WB is extended with a conserved Asp at the +3 downstream location (in NLRP4, 8, 9 and 13 the Asp is substituted by Glu) and is thought to contribute to ATP hydrolysis properties [84].

**Figure 3:**
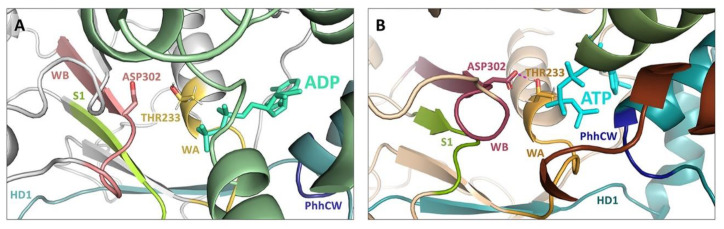
Molecular dynamics simulations of NLRP3 indicate key residues of the ATP-hydrolysing Walker B motif interact with ATP but not ADP. A critical hydrogen bond between the NLRP3 Walker A (WA) and Walker B (WB) positions the two motifs suitably for hydrolysis of the bound ATP (Thr233 and Asp302, respectively). In (**A**) the 3.8 Å cryo-EM structure of NLRP3 (PDB: 6NPY) is ADP-bound, and the residues are at a distance of 4.8 Å, exceeding the limit for hydrogen bond activity. However, as shown by a representative snapshot in (**B**) a 10 ns molecular dynamics simulation of NLRP3 with ATP yielded an average distance of 1.75 Å between the side chain Thr hydroxyl group in WA and Asp carboxyl group in WB, consistent with the formation of a hydrogen bond.

A recent structural model of NLRP3 with ADP occupancy does not display a hydrogen bond between the first Asp residue of the WB motif and the conserved Thr of the WA thought to be essential for proper relative positioning of the two Walker motifs needed for ATP hydrolysis [76]. Conversely, a 10 ns molecular dynamics simulation of NLRP3 with ATP yielded an average distance of approximately 1.75 Å between the hydroxyl group of the Thr side chain of WA and the carboxyl group of the Asp side chain of WB, consistent with the formation of a hydrogen bond (Figure 3). This suggests that the close proximity of the WA and WB motifs is driven by the binding of the nucleotide within the catalytic site.

The downstream S1 motif is proximal to the WA and WB motifs and contains a conserved Arg sidechain that contacts the γ-phosphate of ATP. The basic residue coordinates and transmits the structural reorganization of the NBD upon “sensing” ATP in the active site. With the exception of NLRC4, NLRP4, NLRP9 and NLRP13, all NLR family members contain this Arg residue [78].

STAND ATPases typically contain a conserved helix within the HD1 at the C-terminus of the NACHT domain. The helix contains a Gly residue, followed by any residue and an important proline. In NLRs the glycine is lacking although the Pro residue within the PhhCW consensus sequence is highly conserved throughout the family (Figure 1A). The Pro sidechain (Pro412 in NLRP3; Figure 1A, Figure 2C,D) interacts with and stabilizes the adenine moiety of the bound nucleotide. This is corroborated by models of NLRP3 and NLRC4, and the topology is expected to be retained in all NLRs with the exception of NLRP11 [15,76].

Notably, while AAA+ ATPases typically contain a Sensor 2 (S2) motif located downstream of S1 that completes the active site of the neighbouring protomer, it is largely absent in the STAND class of enzymes and has not been functionally characterized in NLRs. Ostensibly, the coordination of the nucleotide is substituted in NLRs by a conserved His residue (His520 in NLRP3, Figure 1A, Figure 2C,D) in the Winged Helix Domain (WHD), proposed to coordinate nucleotide binding, hydrolysis, and conformational changes between subunits [155]. This His residue is part of a conserved FxHxxxQEhxA sequence found in WHDs of NLR proteins, described as unique and likely to substitute for the S2 functionality in the NLRs [13,78]. NLRP6, NLRP8, NLRX1, CIITA and NAIP are exceptions and do not retain the His; this function is likely to be replaced by another molecular feature since NAIP, NLRP6 and NLRP8 display ATP-binding properties or require ATP for activation. Murine orthologs of human NAIP (NAIP2 and NAIP5) are known to bind ATP via structural analyses. A single mutation in the NBD of NAIP (Lys476 of the WA) disrupted effective inhibition of procaspase-9 and procaspase-3 cleavage, and its interaction with caspase-9 occurred only in the presence of ATP [15,93,156,157,158]. A recent study of NLRP6 provided some evidence for ATP-binding properties; LPS-induced NLRP6 dimers were assembled into higher molecular oligomers only in the presence of ATP [139]. Additionally, both NLRP6 and NLRP8 could be captured with immobilized γ-linked ATP-Sepharose and eluted in a concentration-dependent manner with ATP [80].

Finally, the HD2 domain interacts extensively with the NBD and the N-terminal region of the LRR domain. In conjunction with the LRR, HD2 is known to negatively regulate the function of the NLRC4 α-8 helix located at the C-terminus of the NBD, downstream of the S1 [15]. Constitutive activation of LRR deletion mutants propelled the pervasive characterization of LRR domains as the primary ligand sensing regions, but in more substantive investigations of NAIP-NLRC4 inflammasomes that employed chimeric NAIPs, the ligand specificity was mainly governed by NBD-associated domains [91]. Deletion of the HD2 in murine NLRC4 resulted in an increased release of IL-1β that was more efficient than NLRC4ΔLRR, which was augmented when compered with wild-type NLRC4. These experiments support a critical role for the HD2 over and above the LRR in autoinhibition via steric masking of the α-8 helix. In NLRP3, the HD2 is imperative for NEK7 binding, which was deemed to be essential for NLRP3-inflammasome activation [76].

Taken as a whole, the enzymatic properties of NBD are reliant upon both Walker motifs. Indeed, many studies have shown that mutations within the critical WA and WB motifs either significantly reduce or completely inhibit the biological function of NLRs (and STAND AAA+ ATPases in general). The S1, HD1, WHD and HD2 motifs make stabilizing contacts with each other and provide important intramolecular interactions with the NBD to facilitate the nucleotide binding and hydrolysis properties of the WA and WB motifs, respectively. The WHD is critical for auto-inhibition of enzymatic activity; it stabilizes the closed, inactive conformation of NLRC4, and mutations to this motif are associated with constitutive activation of inflammasomes. Based on the NLRC4 crystal structure, the HD2 motif appears to repress activity by contacting a conserved and important α-helix of the NBD. During activation of the NLRC4 protein, the NBD and HD1 rotate 90° relative to the WHD and HD2, creating an open conformation for the NACHT domain to permit self-oligomerization where otherwise there would be steric contacts [15].

## 5. Structural Basis for Inflammasome Assembly Mechanisms

Comprehensive structural characterizations of STAND ATPases APAF-1, Drosophila DARK and *C. elegans* CED-4 were the historical basis for the development of structural models of NLR inflammasome activation, termed the “STAND binary switch” [12]. First, the apo form of the sensor domain stabilizes the basal state of the protein through extensive interactions with the NBD. Inducer-binding then triggers a global conformational rearrangement which removes the inhibiting effect of the sensor domain on the NBD. This allows for a conformational shift in the closed, ADP-bound form to a transient, nucleotide-free intermediate. The transient state is likely short-lived, given the high affinities of characterized STAND ATPases for nucleotides, and high intracellular concentrations of ADP and ATP. Next, ATP binding triggers a structural reorganization that disrupts both inter- and intra-molecular interactions of the NBD and sensor/effector domains which stabilize the “off” conformation and ultimately displaces the WHD away from the bound nucleotide in the NBD. Finally, with the NBD and HD1 multimerization interface solvent exposed, activation can occur via co-factor recruitment and oligomer stabilization with ATP bound at the interface of neighbouring protomers.

Emerging data on NLR-inflammasome nucleation, domain organization and reorganization have provided insights that discern NLRs from other STAND proteins [3,12,70]. In 2007, a low-resolution cryo-EM structure of NLRP1 was the first to emphasize the structural importance of the NACHT domain in NLR oligomerization [35]. The focus on structural analyses of the NACHT domain was subsequently protracted by the self-aggregation tendencies of NLRs, and the NACHT domain in particular, and delayed the procurement of high-resolution NLR structures until recently.

In the intervening period, many researchers conducted both structural and in silico analyses of NLR effector and adaptor interactions. Several structure-based cryo-electron microscopy (EM) studies provided insight into the shared, prion-like polymerization of various effector molecules recruited to activated NLR inflammasomes [140,159,160]. Notable studies of the PYD and CARD domains have revealed both to form filamentous assemblies in the nucleation of inflammasome complexes [140,161,162]. The effects of inducer binding in sensor domains were surveyed computationally, including NOD1 LRR interactions with IF-DAP [46], NOD1 and NOD2 CARD with RIP2 [163,164], and NOD2 MDP binding [165]. The CARD-CARD interactions of NOD2 were also examined [165], and additional reports on effector domains have included NLRP3-POP1 PYD-PYD interaction dynamics [166], NLRP1/Caspase-1 CARD-CARD interactions [167], and the conformational dynamics and fold stability of NLRP PYDs [168]. In summary, these domains mediate the homotypic interactions critical for the assembly of filamentous NLR inflammasomes. NLRs were found to nucleate PYD filaments of ASC, which in turn clustered the CARD of ASC, and finally nucleated CARD filaments of caspase-1. Additionally, many groups have highlighted or suggested important structural and spatial arrangements in the central ATP-binding NACHT domain that contribute to inflammasome nucleation and signalling. While these studies contributed to our current understanding of inflammasome effector recruitment and adaptor regulatory mechanisms, empirical structural descriptions of NLRC4 and APAF-1, NLR enzymology analyses, and modeling of the NACHT domain in particular, established the importance of ATP-driven conformational changes now known to be essential for modulating the protein-protein interactions that were first surveyed.

**Figure 4:**
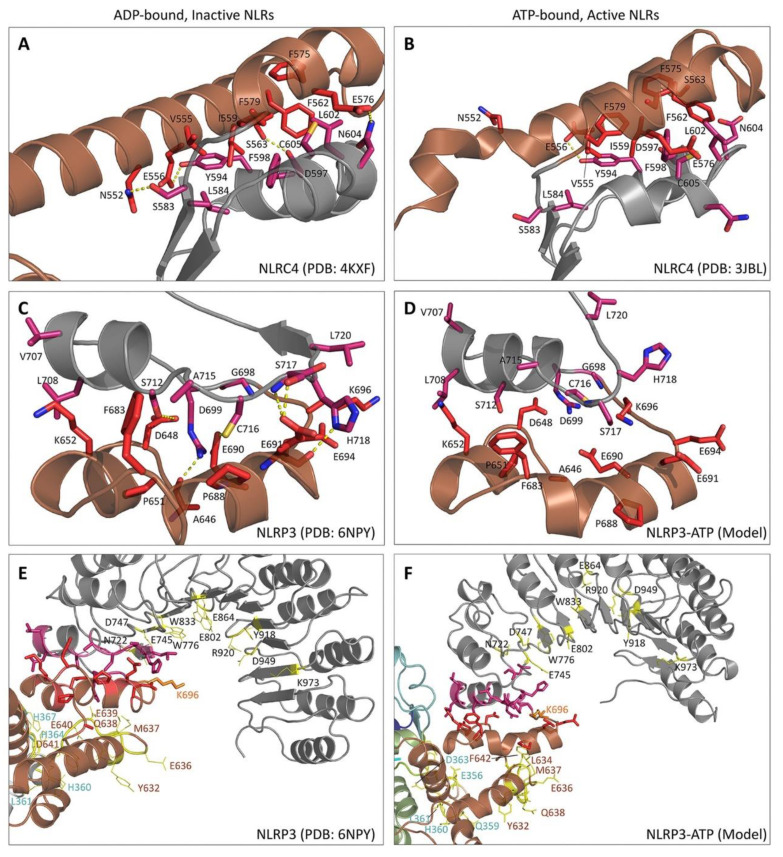
HD2-LRR interactions within NLRC4 and NLRP3 proteins. The detailed interaction interfaces are highlighted for the helical domain 2 (HD2, brown) and the N-terminal region of the leucine rich repeat (LRR) domain (gray). The side chains from the HD2 and LRR are coloured in pink and red respectively. Dashed yellow lines indicate hydrogen bonds. (**A**) ADP-bound NLRC4 at 3.2 Å resolution [15], (**B**) ATP-bound NLRC4 at 4.7 Å resolution [89], (**C**) ADP-bound NLRP3 at 3.8 Å resolution [73], and (**D**) representative ATP-bound NLRP3. Overview of NEK7 interfaces in NLRP3-ADP (**E**) or NLRP3-ATP (**F**). NEK7-binding residues are shown in yellow, or orange if overlap occurs with the HD2-LRR interface. In (**E**,**F**), labels are colour coded with respect to motifs.

The first crystal structure of an NLR-NACHT domain was published in 2013 [15]; it represented a closed, ADP-bound 3.2 Å resolution structure of murine NLRC4 (PDB ID: 4KXF). The general topology of the NLRC4-NBD was similar to other STAND proteins. It contained a three-layered α/β structure; however, an additional β hairpin was located upstream of the WA motif and was shown to interact with the LRR. The LRR functioned to sterically occlude one side of the NBD and maintain the monomeric state of inactive NLRC4. This mechanism of autoinhibition was supported by constitutive activation of the NLRC4 inflammasome when ADP-mediated NBD-WHD/-HD2 or -LRR interactions were disrupted (Figure 4A).

Bound-ADP in the NBD was critical for the stability of the inactive conformation of NLRC4, as observed previously for APAF-1. The phosphate groups of ADP act to stabilize the WA motif with five hydrogen bonds, and the guanine base of ADP provided two additional bonds to the NBD. In APAF-1, the conserved His of the WHD (His438) interacted specifically with the β-phosphate group of ADP but not ATP, and the NBD and HD1 were fixed together by the presence of ADP in the active site [169,170]. This critical His residue was conserved in NLRC4 (His443) and mediated the NBD and WHD interaction for stabilization of the closed conformation of NLRC4. The conservation of key ADP-WHD interactions in ADP-bound NLRC4 (including the key His residue), plus the comparable positioning of the WHD/NBD, suggested a similar mechanism of action in NLRC4. ADP to ATP exchange in the APAF-1 NBD instigated a structural remodel of the WHD with respect to the NBD, disrupting the interaction with ADP. Mutation of His443 to Leu resulted in constitutive activation of IL-1β and increased oligomer formation in HEK293T cell inflammasome reconstitution systems. This points to the importance of this interaction with ADP in obstructing conformational changes in the WHD that lead to ADP unbinding and activation.

Indeed, when active structures of NLRC4 were published in 2015, superposition with the inactive structure revealed the WHD-HD2-LRR module to rotate 87.5° along an axis at the junction between HD1 and WHD, rearranging the intramolecular interactions between the WHD and NBD [93,94]. These studies illuminated a mechanism of sequential ligand-induced assembly for the NLRC4 inflammasome, a process in which ligand binding first activated NLRC4 to induce the structural reorganization of the WHD-HD2-LRR and promote the exchange of ADP for ATP. Subsequently, a catalytic surface was exposed that could activate a second inactive NLRC4 molecule, thus self-propagating the active conformation and initiating assembly of the inflammasome complex. This required a concerted reorientation of the WHD from the NBD in the inactive state, to avoid clashes between the β-hairpin tip of WHD from an activated NLRC4 and the receptor surface of an inactive NLRC4.

A conflicting publication by Diebolder and co-authors suggested the ATP-bound conformation of NLRC4 did not possess rigid body movement between HD1 and WHD, but instead observed a 21˚ rotation of the NBD-HD1 segment and a 49˚ rotation of the LRR domain with respect to the WHD-HD2 module [92]. The authors employed cryoelectron tomography (cryo-ET) and subtomogram averaging to obtain a higher-resolution structure; they concluded conformational changes within the NACHT domain and rotation of the entire LRR domain as a distinct rigid body were required to establish the intermolecular interactions necessary for inflammasome activation.

Although mechanistic disparities with respect to module rearrangement arose in these investigations, the key function of bound-ATP in stabilizing the ligand-induced active state was non-controversial. Allosteric activation via ligand binding induces conformational changes in the nucleotide-binding site that are transmitted globally to disengage the autoinhibitory domains from the NBD and render it accessible to nucleotide exchange and successive oligomerization. This role was confirmed in analyses of APAF-1, where the NBD and HD1 were held in close proximity by ADP, and inducer-promoted activation elicited a rotation of the WHD-HD2 module to destabilize interactions of ADP to the NBD and allow for nucleotide exchange followed by apoptosome activation [169,170].

Interestingly, while bound-ATP functioned to stabilize the active conformation of APAF-1 through various interactions, ATP bound in the NLRC4 inflammasome did not directly contribute to the stabilization of the active structure and only interacted with the NDB and HD1. Additionally, mutations in the P-loop of NAIP5 suggested that ATP-binding was dispensable for the assembly of the NAIP5-NLRC4 inflammasome [171]. While early data alluded to a central role for the ATPase activity of APAF-1 in activation, the non-hydrolyzable ATP analog AppNHp was sufficient to support assembly of apoptosome, highlighting the stabilizing role of ATP in the “on” state of APAF-1 [172]. Importantly, inactive APAF-1 but not APAF-1 from the apoptosome displayed low ATPase activity. Amongst other NLR members, variable requirements for ATPase activity requirements have been reported. For example, mutations in the P-loop of NLRP1 resulted in constitutive activation [102] while mutations of the corresponding residues in NLRP3 appreciably attenuated inflammasome activity [45].

In computational modeling, Afanasyeva and colleagues reported a WHD-NBD opening mechanism for ATP-bound TIP49 (a non-NLR AAA+ ATPase) during a 30 ns molecular dynamic simulation run [173]. In contrast, Maharana and colleagues failed to display the opening mechanism of ATP-bound NOD1/NOD2-NACHT domains in a zebrafish model for 50 ns of MD simulation [46]. In longer duration, 200 ns simulations with NLRP1-, NLRP3- and NLRC4-NACHT-ATP-Mg^2+^ bound homodimeric complexes modeled on the NBD-HD1 of NLRC4, no significant rigid body movement was detected between HD1 and WHD when compared against ADP-bound models [47]. The authors concluded the interaction of ATP-Mg^2+^ might not be the only component to drive the switching mechanism in NLRs and suggested PAMP/DAMP recognition to play a more significant role than nucleotide exchange.

In 2019, Sharif and colleagues published a 3.8 Å cryo-EM structure of the NLRP3ΔPYD and mitotic kinase NEK7 complex and expounded upon the molecular mechanism of NEK7-mediated activation of the NLRP3 inflammasome [76]. The reported cryo-EM structure was akin to the earring shape, or “inverted question mark” structure of NLRC4 [15,93,94], NOD2 [174] and NAIP5 [156,175] with a curved LRR and globular NACHT domain (Figure 2A). The structural model revealed that NEK7 contributed significantly in a scaffolding role, interacting electrostatically with the HD2, NBD and LRR domains of NLRP3 and bridging the gaps between adjacent NLRP3 protomers in NLRP3 oligomerization. The NBD–HD1–WHD module of NLRP3 displayed comparable positioning and conformation to the inactive structures of NLRC4 and NOD2, but the HD2 and LRR domain topologies were distinct among the other structurally-characterized NLRs [15,174]. The aforementioned studies on NLRC4 have indicated the NACHT domain undergoes a large rigid-body rotation at the HD1-to-WHD junction upon ligand binding to open the structure for nucleotide exchange, oligomerization and activation. A hypothetical structure of NLRP3 in an active conformation, modeled on the NLRC4 oligomer structure as a reference, generated a comparative ~90° rotation of the NBD-HD1 module. This positioned the activated NBD-HD1- WHD module for direct interaction in the NLRP3 ring with no steric hindrance; however, the inactive conformation was not compatible with the multimeric complex. The hypothetical active-NLRP3–NEK7 inflammasome disc placed NEK7 at the oligomerization interface. The NLRP3-LRR was not large enough to contact the adjacent LRR in the oligomer model, and thus NEK7 bridged the gap between adjacent protomers by projecting outward from the plane of the inflammasome ring and relieving interference during NLRP3 oligomerization.

As previously discussed, autoinhibition of NLRs via sensor domain-NBD interfacing is well supported in the structural models of APAF-1 and NLRC4/NAIP inflammasomes. The interactions of NEK7 with the HD2, NBD and LRR domains of NLRP3 are interesting in that some overlap with LRR-HD2 articulation motifs or are proximal to key stabilization motifs (Figure 4C,D). The network of hydrophobic interactions and hydrogen bonds between the HD2 and LRR domains could indicate a similar mode of autoinhibition as observed for NLRC4 (Figure 4A,B). However, the function of the LRR domain in NLRP3 autoinhibition and/or activation is a matter of some debate, with several contradictory functions and regulatory mechanisms disseminating. The NEK7-NLRP3 structure was coordinated by an expansive network of interactions within the LRRs, and biochemical profiling of NEK7-NLRP3 complex formation has indicated an indispensable requirement of the LRR in NEK7-NLRP3 binding [76,125]. Conversely, another study has examined LRR-deletion mutants and concluded that the LRR did not provide autoinhibition as several truncated LRR domain mutants could bind effectors, initiate polymerization and reconstitute disease in *Nlrp3*^−/−^ mice [176]. Further investigations are required, as other studies have demonstrated the potential for NLRP3-LRR to negatively regulate inflammasome activation [113,140,177,178,179].

Both NEK7-binding and the NACHT conformational change induced by ATP-exchange are obligate for NLRP3 activation [125]. NEK7 was required for caspase-1 processing and IL-1β release in murine macrophages containing either wild-type or hyperactive CAPS-associated mutant NLRP3-R258W (human NLRP3-R260W), indicating NEK7 regulates the inflammasome at or just downstream of NLRP3. This result held for inflammasome induction for a wide range of stimuli including ATP, nigericin, toxin gramicidin, particulate matter, the lysosome membrane damaging agent Leu-Leu-OMe, and cytosolic LPS stimulation that activates the non-canonical inflammasome via caspase-11.

Both Sharif et al. [76] and He et al. [125] have proposed a similar activation mechanism whereby NLRP3 must be both associated with NEK7 (induced by potassium efflux) and have undergone ATP-exchange and conformational transition prior to oligomerization, although the specific order in which those steps occur remains unclear [125]. While mechanisms of autoinhibition in other NLRs point to the likelihood of inducer-binding preceding ATP-exchange, whether those stages involve other inducers, post-translational modifications, cellular re-localization and other regulatory measures is not only probable, but presumed. However, these structural data for NLRP3 will be obligate for the structure-function studies needed to address current knowledge gaps.

**Figure 5:**
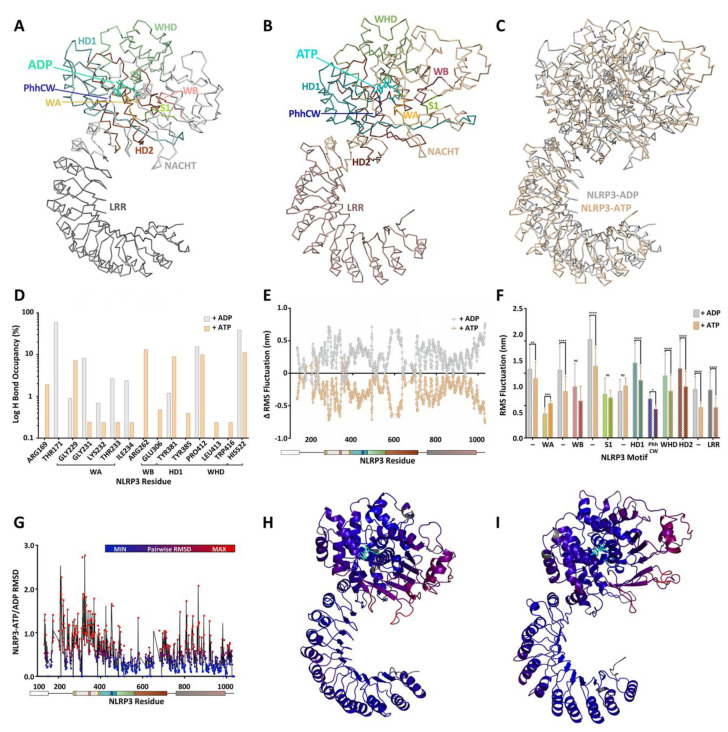
Binding of ATP or ADP ligands to NLRP3 suggests holistic structural differences. Backbone images are provided for the NLRP3-ADP (**A**) and NLRP3-ATP (**B**) structures. Important domains and motifs are colour-coded and labelled following visual rendering with PyMOL v2.4 (pymol.org), and the bound nucleotide ligand is shown in stick rendering. In (**C**), the backbone structures of NLRP3-ADP and NLRP3-ATP were aligned by sequence. (**D**) Hydrogen bond occupancy between NLRP3 and ADP or ATP observed over the course of 10 ns molecular simulation. The plot indicates how often hydrogen bonds between the nucleotides and the protein were observed during the simulation. (**E**) Root-mean-square (RMS) fluctuations of NLRP3 residue positions were calculated during the 10 ns molecular simulations with ADP or ATP. The average RMS fluctuations (nm) were plotted along the length of the NLRP3 primary sequence. Note: the PYD was not present in the NLRP3 protein construct used to generate the structure of the 6NPY deposition. (**F**) The average RMS fluctuations were calculated for each of the important NLRP3-NACHT motifs involved in nucleotide-binding and hydrolysis. The unstructured linker sequences connecting the domains/motifs are indicated by (—). Significantly different between ADP-and ATP-bound forms of NLRP3-NACHT: *, *p* < 0.05, **, *p* < 0.01, ***, *p* < 0.001, and ****, *p* < 0.0001; two-way ANOVA with Holm-Sidak *post hoc* test. In (**G**), the NLRP3-ATP and NLRP3-ADP structures were compared across the 10 ns molecular simulations, and RMSD values were calculated for all the NLRP3 residues and plotted along the length of the NLRP3 primary sequence. The RMSD values were used to colour the NLRP3-ADP (**H**) and NLRP3-ATP (**I**) ribbon diagrams. The structures are coloured with heat-mapping, where blue to red, where red designates those residues which fluctuated most between the ADP and ATP bound structures (highest RMSD values).

We employed molecular dynamics (MD) simulations of ADP- and ATP-bound structures of NLRP3 to examine the global effects on protein structure dictated by the bound nucleotide. MD simulations were carried out to assess the dynamic stability and structural behavior of the ADP-bound cryo-EM structure (PDB: 6NPY with 3.80 Å resolution [76]) and an ATP-bound model in which the additional γ-phosphate was artificially modeled with Chimera [180]. The data trajectory files for the MD simulations were collected over a simulation time period of 10 ns (Methods S1; [181,182,183,184,185]), attaining stable conformations in less than 8.5 ns. These simulations revealed distinctions in global protein structure, catalytic site conformation and hydrogen bond configuration (Figure 5A–C; Videos S1 and S2). The representative ATP-bound structure demonstrates a 36˚ rotation of the LRR with respect to the WHD-HD2 module when compared with the empirical ADP structure. This topology is reminiscent of the indispensable rotation associated with the WHD-HD2 in APAF1 that was necessary for nucleotide exchange and activation [169,170]. Upon comparing the two structures, the ADP-bound interface between the WHD and the LRR is characterized by a high degree of closure when compared with that of the ATP-bound model. ADP release from the closed-ADP interface is very likely contingent on a separate event in an adjacent packing unit, such as inducer sensing or cofactor binding (e.g., NEK7 binding). Unlike APAF1, the in vitro application of ATP or ATP analogues to inactive-state NLRP3 does not induce a conformational change or oligomerization, and the tight packing at this interface could account for this finding [76].

Structural changes in the active site of the ADP- and ATP-bound models were analysed, and the number of formed hydrogen bonds was determined for each (Figure 5D). Four active site residues, Pro412, Leu413, Tyr381 and His522 provide stabilizing contacts to both bound nucleotides, while conformational shifts in conserved nucleotide-interacting motifs provide variable hydrogen bonds to ADP and ATP. Ultimately, these distinctions reflect the altered structural effects of ADP or ATP occupancy on the global protein structure. ADP is critical for the stability of the inactive conformation of NLRC4 and APAF1, and the five hydrogen bonds coordinating ADP-binding are conserved in the ADP-bound structure and throughout the trajectory [15].

The change per-residue root mean square fluctuations (ΔRMSF) results demonstrate the change in flexibility of all the backbone Cα atoms of the amino acid residues for NLRP3 with the binding of ADP or ATP. The ΔRMSF values were calculated as the difference between ATP-bound and ADP-bound states, and the difference between the ADP-bound and ATP-bound states (Figure 5E). ATP-bound NLRP3 exhibits an overall loss in protein flexibility, as can be observed by the negative peaks. Remarkably, ATP binding increases flexibility in less than 1% of total residues, yet increases the apparent flexibility in the entirety of the WA motif (residues 226–233), 4 of the 6 residues in S1 (348–351), residues 365–370 of the HD1, residues 509–513 of the WHD, and residues 640–642 and 697 of the HD2 motifs. This highlights the importance of residues within these motifs in coordinating the nucleotide and contributing to the structural reorganization that occurs following the “sensing” of ATP in the active site. Merged data for each motif is shown in Figure 5F. The ADP- and ATP-bound structures share an overall RMSD of 0.85 Å, ranging in distance from 0.10 Å to 1.79 Å over the course of the simulation trajectories. These data are shown in Figure 5G, with regions of highest energy deviation projected onto the structures in red (Figure 5H,I).

NLRC4 has well defined LRR-HD2 interactions that are known to function in an autoinhibitory fashion at basal state (Figure 4A,B). Comparative analyses of the ADP- and ATP-bound structures of NLRP3 reflect similar positioning of the LRR and the HD2 (Figure 4C,D). This could suggest the NLRP3-LRR functions in a similar fashion to NLRC4, preventing conformational reorganization, nucleotide exchange and activation. This would necessitate the removal of the inhibitory effect, likely initiated by inducer binding prior to nucleotide exchange and conformational rearrangement, as displayed in the active, ATP bound NLRC4 structure (Figure 4B). The ATP-bound NLRP3 model undergoes a comparative disruption of hydrogen bonding networks and close packing of helices, again suggesting a similar mode of action (Figure 4D). Additionally, an essential cofactor in NLRP3 inflammasome activation is NEK7 [76,125], which has a defined interaction interface containing residues involved in the putative auto-inhibitory interface (Figure 4E). Interestingly, the ATP-bound NLRP3 state has reorganized such that the residues which make up the NEK7 interaction interface become accessible for binding (Figure 4F).

Ultimately, these MD simulations and comparative analyses suggest a tightly packed “closed” ADP-bound structure for NLRP3 that undergoes a large-scale domain rotation to “open” in the ATP-bound form. If the closed structure inhibits nucleotide exchange, the removal of the inhibiting effect via inducer or cofactor binding would be expected to precede the conformational changes necessary for activation.

## 6. Pharmacological Inhibitors of NLRP3 ATPase Activity

**Figure 6:**
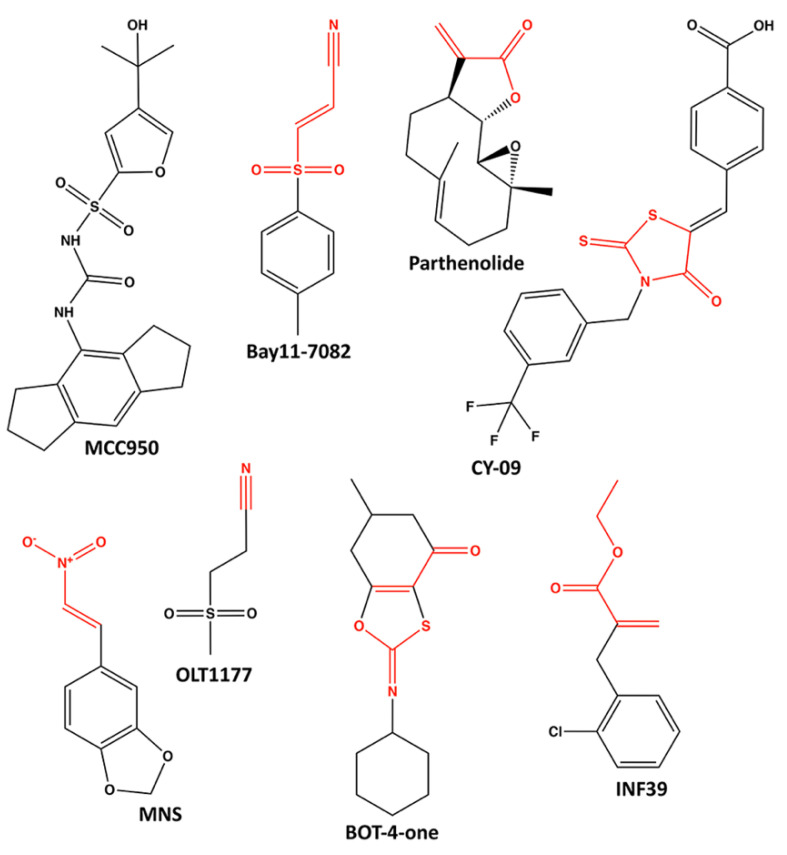
Small molecule inhibitors targeting the ATPase Activity of NLRP3. Chemical structures of NLRP3 Inhibitors which target the ATPase activity of NLRP3 are provided. The molecular structures were generated in ChemDraw v19, and the Michael acceptor moiety is coloured in red for electrophilic inhibitors. PubChem Compound Identification Numbers: MCC950, 9910393; parthenolide, 7251185; Bay11-7082, 5353431; CY-09, 75070350; MNS, 672296; OLT1177, 12714644; Bot-4-one, 16129399; and INF39, 69150705.

While NLR inflammasomes are mainly triggered by exogenous PAMPs, NLRP3 can be triggered by sterile, endogenous or environmental DAMPs signals. The causative role of NLRP3 in innate immune responses associated with human health and the progression of autoinflammatory and autoimmune diseases has compelled a growing interest in the pharmacological mediation of NLRP3-associated inflammation [186,187,188]. The complex signaling networks which contribute to its activation have convoluted the identification of specific NLRP3 inhibitors. Compounds which neutralize the production of inflammatory cytokines produced by the NLRP3 inflammasome are not necessarily specific or direct, and could impact on upstream signaling events (e.g., priming or effector recruitment) or downstream signaling events (e.g., caspase-1 activity, gasdermin D cleavage, or pore formation). Several NLRP3 inhibitors are now understood to have multiple off-target effects, including those acting on anti-inflammatory components of innate immunity or on other components and products of the inflammasome. Direct inhibitors are thus considered to be promising pursuits for anti-inflammatory therapies. Moreover, these molecules are expected to offer important insight into the regulation of NLRs, particularly those which directly inhibit the ATPase activity. Herein, we provide a brief overview of compounds that have been characterized to possess inhibitory potential toward NLRP3 ATPase activity, including MCC950, Bay11-7082, parthenolide, CY-09, MNS, OLT1177, BOT-4-one and INF39 (Figure 6).

### 6.1. MCC950

A well-established inhibitor of NLRP3 inflammasome signaling, glyburide, was identified in 2001 to attenuate IL-1β processing and release following NLRP3-inflammasome stimulation [189]. Deemed to be selective since it did not impair NLRC4 or NLRP1 signaling, the sulfonylurea derivative worked upstream of the NLRP3-inflammasome and downstream of the ATP receptor P2X7 [190]. Though indirect, the potency of glyburide for NLRP3 led to enthusiastic research on sulfonylurea derivatives and to the discovery of MCC950- a selective and direct inhibitor of NLRP3 that did not impact AIM2, NLRC4, or NLRP1 inflammasome signaling [191].

MCC950 is a diarylsulfonylurea-containing compound that suppresses ATP hydrolysis through direct binding of the ATP binding region, likely within the Walker B motif [192,193]. MCC950 does not suppress NLRP3 activity by targeting inflammasome priming, calcium signaling, potassium efflux or NLRP3-ASC binding [191]. The compound can inhibit K^+^ efflux-independent NLRP3 activation but not mitochondrial respiration or ROS production [194,195].

Coll and colleagues employed a drug affinity responsive target stability (DARTS) approach with mouse bone marrow-derived macrophages and human monocyte-derived macrophages with the broad specificity protease mix, pronase, to induce degradation of NLRP3 [192]. Increasing doses of MCC950 was protective toward pronase-mediated NLRP3 degradation and was specific for NLRP3, while having no impact on NEK7 or GAPDH degradation. Moreover, MCC950 protected NLRP3 from degradation in both its inactive and nigericin-stimulated active conformations. Using truncation and missense mutants, the authors localized the binding site of MCC950 to the NACHT domain, specifically within or proximal to the Walker B region. Interestingly, a chimeric NLRP3 mutant containing the NLRP12-NACHT domain was not protected by MCC950 in the degradation assay, demonstrating the specificity of this compound for NLRP3 even within the NLRP family. Considering the high level of conservation within the Walker B sites of NLRP3 and NLRP12 (Figure 1A), with only a single conservative variance in the first position (Ile296 in NLRP3, Leu288 in NLRP12), differential mechanisms of regulation and conformational status likely drive this distinction. MCC950 provides reversible NLRP3 inhibition; surface plasmon resonance (SPR) assays examining the interaction kinetics of MCC950 with NLRP3 revealed high-affinity binding with a rapid off-rate. MCC950 did not compete with ATP for NLRP3 binding but led to a stable interaction for both the inhibitor and nucleotide, with a lack of ATP hydrolysis in the presence of MCC950.

To examine the effect of MCC950 on intramolecular conformational shifts, Tapia-Abellàn and colleagues employed a bioluminescence resonance energy transfer (BRET)-based proximity assay to demonstrate that wild-type NLRP3 and the disease-associated hyperactive mutant D305N-NLRP3 have distinct conformations, wherein the amino- and carboxyl-termini of the hyperactive NLRP3 are separated as compared to the wild-type topology [193]. This open conformation was also observed for other hyperactive disease mutants and was not impacted by variations in protein expression levels. Nigericin-induced NLRP3 oligomerization coincided with increased inflammasome signaling and stabilization of an NLRP3 open structure, suggesting that NLRP3-activating stimuli drive a similar open and active NLRP3 conformation as that observed for the hyperactive disease mutants. In HEK293T cells where overexpressed inflammasome components NLRP3, ASC and CASP-1 spontaneously form inflammasomes, MCC950 could elicit near-complete disaggregation of active oligomers. This held true for WT-NLRP3 as well as hyperactive mutants R262W, D305N, and T350M. Incubation of both the D305N disease mutant and wild-type NLRP3 with MCC950 resulted in a loss of IL-1β processing with a corresponding dose- and time-dependent increase of signal for a closed conformation. This MCC950-dependent abrogation of conformational rearrangement was reversible. The results suggest that MCC950 can bind and interfere with conformational changes in both resting as well as activated and oligomerized forms.

Ultimately, MCC950 is a promising therapeutic agent that directly binds to NLRP3 and prevents structural rearrangement or retention of the open conformation observed in stimulated WT and hyperactive mutant NLRP3. This mechanism of interference in conformational rearrangement could provide fascinating insight into the role of structural shifts in the activation scheme of NLRP3.

### 6.2. Parthenolide and Bay11-7082

Parthenolide, a plant sesquiterpene lactone isolated from *Tanacetum parthenium* and Bay11-7082, a synthetic vinyl sulfone discovered in 1968, could prevent NLRP3 signaling and the release of IL-1β from PMA-differentiated and ATP-stimulated THP- 1 cells [196]. Mechanistically, parthenolide provided alkylation of cysteine residues of caspase-1 in response to NLRP1, NLRC4, and NLRP3 stimulation, and Bay11-7082 targeted the NF-κB pathway via alkylation of essential nucleophilic residues of IKKβ. Both vinyl sulfones could inhibit the ATPase activity of NLRP3 in vitro and demonstrate direct interaction under those conditions; however, they are each broad-spectrum inhibitors active against multiple targets in inflammatory responses [196,197].

Both compounds are irreversible covalent inhibitors. Each electrophilic derivative contains a Michael acceptor moiety that is highly reactive to nucleophilic residues in proteins (e.g., cysteine side-chains). Although Michael acceptors are often characterized as indiscriminate, the targeted covalent modification of explicit cysteine residues has emerged as a validated approach to drug discovery with several electrophilic acrylamide-containing drugs recently obtaining FDA approval [198]. Vinyl sulfone derivatives have been effective and well-tolerated as antiparasitic agents in dogs and mice [199], and permeate cell membranes easily [200]. Despite the unselective nature of these compounds, the direct targeting of NLRP3 ATPase activity provided a basis for future success in the development of covalent and irreversible electrophilic inhibitors for therapeutic mediation of NLRP3 activity [201].

### 6.3. CY-09

In 2017, Jiang and colleagues specifically targeted the ATPase activity of NLRP3 as a potential mechanism for the treatment of NLRP3-associated diseases [202]. In a screen of NLRP3 inhibitors, they found that CFTR(inh)-172 (i.e., an inhibitor for the cystic fibrosis transmembrane conductance regulator (CFTR) channel [203]), could block NLRP3 inflammasome activation. An analog that lacked CFTR-inhibitory activity, CY-09, specifically bound the WA motif of NLRP3 to inhibit ATP binding and hydrolysis and suppress inflammasome activation. CY-09 was found to block ATP-, MSU- and nigericin-induced activation of the NLRP3 inflammasome and cytokine maturation without affecting the signaling of NLRC4, NLRP1, NOD2, or RIG-I inflammasomes.

A biotinylated analogue of CY-09 could pull down NLRP3 but not other inflammasome components (including ASC, caspase-1 or NEK7), and the interaction could be competed with free CY-09. A microscale thermophoresis (MST) assay was used to provide dissociation constant (K_D_) of ~500 nM for CY-09 and purified GFP-NLRP3. CY-09 could inhibit the ATPase activity of NLRP3 with an IC_50_ value of 5 μM, but no effect was observed against the ATPase activity of NLRC4, NLRP1, NOD2 or RIG-I. In mutational analyses, CY-09 could bind a Walker B mutant of NLRP3 while mutation of the WA motif impeded binding. Correspondingly, CY-09 could compete bound ATP from NLRP3 in a concentration-dependent manner. The results suggest that CY-09 blocks ATP-binding via direct competition for the WA motif and thus eliminates ATPase activity. This was further supported by computational analysis, with modeling inferring that CY-09 was readily docked to the WA motif. Taken together, the current literatures suggets that CY-09 is a selective NLRP3 inhibitor with an appropriate pharmacokinetic profile to provide therapeutic effect in murine models of disease.

### 6.4. 3,4-Methylenedioxy-β-Nitrostyrene (MNS)

3,4-Methylenedioxy-β-nitrostyrene (MNS) was discovered in a kinase inhibitor screen by He and colleagues in 2014. The molecule provided selective inhibition of NLRP3 oligomerization with no effect on the AIM2 or NLRC4 inflammasomes [204]. In addition, MNS could block ATP-, nigericin- and silica-stimulated NLRP3 activation in LPS-primed BMDMs. It is important to note that MNS also displays inhibitory activity for tyrosine kinases such as Src and Syk [205]. Structure-activity-relationship (SAR) assessments of MNS analogues with modifications on the nitrovinyl or dioxole group of the compound suggested that the nitrovinyl side chain was essential for MNS-mediated inhibition of NLRP3 activity, while the dioxole group was unnecessary. Chemical modifications will be required in order to provide additional specificity for the MNS scaffold toward NLRP3 over tyrosine kinases.

MNS could directly interact with NLRP3, and biotinylated-MNS could pull down NLRP3 as defined by mass-spectrometry and streptavidin-coupled bead precipitation [204]. The addition of excess MNS or Bay11-7082 compound abolished these interactions. The functional activity of MNS appears to be mediated through the direct and concentration-dependent inhibition of NLRP3 ATPase activity. The mechanism of inhibition likely involves an alkylation by nucleophilic attack of reactive residues such as cysteine to form an irreversible, covalent linkage to NLRP3. As such, the targeting mechanism is likely similar to those observed for other covalent and electrophilic inhibitors.

### 6.5. OLT1177 (Dapansutrile)

OLT1177 (dapansutrile) is an orally active β-sulfonyl nitrile molecule derived from glyburide, but lacks the cyclohexylurea moiety group which is responsible for the compound’s effect on insulin secretion [206]. OLT1177 was reported to potently inhibit inflammatory cytokine processing by NLRP3 in models of gout and osteoarthritis [207]. In vitro, OLT1177 reduced NLRP3-mediated IL-1β and IL-18 processing by 50% in LPS-primed and ATP-stimulated J774A.1 macrophages at nanomolar concentrations [208]. OLT1177 had no effect on AIM2 or NLRC4 inflammasome signaling, potassium efflux, or mRNA expression levels of inflammasome components. While OLT1177 can influence IκB kinase activation, additional NF-κB independent mechanisms of NLRP3 inhibition were supplied by OLT1177 [208]. In immunoprecipitation and Fluorescence Resonance Energy Transfer (FRET) assays, OLT1177 abolished NLRP3 associations with ASC and caspase-1. Concomitant decreases in NLRP3 oligomerization and activation were also observed. Finally, OLT1177 could attenuate the ATPase activity of recombinant NLRP3, suggesting a direct mechanism for NLRP3 inhibition [208]. Though statistical significance was not noted in the data, levels of inhibition for both 1 and 10 μM OLT1177 were decreased as compared with 10 μM concentrations of Bay11-7082 and MNS.

### 6.6. BOT-4-One

The benzoxathiole derivative BOT-4-one (2-cyclohexylimino-6-methyl-6,7-dihydro-5H-1,3-benzoxathiol-4-one) is as an anti-inflammatory alkylating agent which targets NF-κB signaling as well as NLRP3 [209]. The kinase domain of IKKβ was identified as the probable target of alkylation by BOT-4-one in the NF-κB pathway, with modeling suggesting the nucleophilic addition reaction occurs on Cys179 of IKKβ [210,211]. While BOT-4-one displayed no inhibitory activity toward AIM2 signaling, modest inhibition of NLRC4-mediated IL-1β and caspase-1 maturation was observed, suggesting potential off-target effects even within the NLR family.

BOT-4-one is also reported to be a direct inhibitor of the NLRP3 inflammasome by abrogating ATPase activity and subsequent oligomerization. BOT-4-one was postulated to act upon NLRP3 via covalent alkylation of cysteine residues. Shim and colleagues confirmed the alkylating activity of BOT-4-one in assays with L-cysteine, an alkylation inhibitor that restored all of the observed NLRP3 inflammasome-mediated signals inhibited by BOT-4-one alone (i.e., pro-IL-1β and pro-caspase-1 maturation and ASC-speck formation [209]). These results were similar to those observed with Bay11-7082, a known alkylating agent with inhibitory effects on NF-κB and NLRP3. Interestingly, the authors found that NLRP3 alkylators, including BOT-4-one, Bay11-7082 and MNS could enhance the ubiquitination level of NLRP3 independently of PKA. The study did not distinguish whether BOT-4-one directly promoted NLRP3 ubiquitination or inhibited the deubiquitination process of NLRP3. However, the conservation of increased ubiquitination in the presence of other alkylating agents, as well as the loss of effect with L-cysteine, suggest alkylation of NLRP3 could drive increased ubiquitination. Since deubiquitination of NLRP3 during priming is essential for activation [85], this effect likely contributes to attenuation of NLRP3 signaling.

### 6.7. INF39

INF39 was revealed after successful tuning of electrophilic α,β-unsaturated derivatives for the development of anti-pyroptotic compounds [212]. The original lead scaffold, INF4E, was identified as a reactive Michael acceptor that irreversibly trapped thiol nucleophiles to abolish both ATP- and nigericin-stimulated NLRP3 signaling in human THP-1 cells. The INF4E acrylate derivatives were found to be less cytotoxic than the historical acrylonitriles applied to NLRP3 inhibition. The success of INF4E and associated covalent warhead compounds in inhibiting caspase-1 processing led to the development of structurally related inhibitors. One compound, INF39 (2,4-dinitrobenzenesulfonic acid), identified by Cocco and colleagues in 2017, displayed strong anti-pyroptotic properties and an IC_50_ value of 10 nM [213]. This irreversible inhibitor could decrease IL-1β release in BMDMs with low cytotoxicity. Employing BRET assays, the authors found that INF39 could block the formation of oligomers, ASC specs, and NLRP3 activation in nigericin-stimulated BMDMs. In their assay, nigericin treatment resulted in a decrease of NLRP3 BRET signal followed by recovery. These fluctuations were inferred to be the result of conformational changes in NLRP3 protein during the activation scheme that result as a consequence of the intracellular K^+^ decrease induced by nigericin since they could be avoided by application of nigericin in high K^+^ buffer. INF39 did not affect the initial drop in BRET signal but was able to impair the recovery of BRET, suggesting that while INF39 could not block the initial conformational changes induced in NLRP3 by decreased intracellular K^+^ concentration, it affected a second, K^+^ independent stage of NLRP3 conformational change that was associated with the ATPase activity. Additionally, INF39 reduced the steady-state (or basal) BRET signal of NLRP3 without affecting the viability of cells after incubation for 24 h. These data imply that INF39 could target the basal NLRP3 conformation as well as activated NLRP3 inflammasomes.

INF39 displays non-specific targeting with broad anti-inflammatory activity toward NF-κB. While INF39 could inhibit NLRP3 priming, the molecule was also found to directly target the ATPase activity of NLRP3. The chemistry of INF39 and expected alkylation mechanism was supported by in vitro observations of covalent, irreversible inhibition. No restoration of NLRP3 ATPase activity was observed after wash-out of INF39 prior to ATP addition, although a control analog showed full recovery of ATPase activity following the wash step. Indeed, earlier in silico work by the same group found a potential target of alkylation by acrylamide at Cys419 within the ATPase catalytic pocket of the NACHT domain of NLRP3 [214].

## 7. Conclusions

Over the past decade, studies to characterize the ATP-binding and ATP-hydrolysis properties of NLRs have provided impactful findings that have ultimately propelled a more complete understanding of inflammasome biology. Biochemical and structural analyses continue to reveal the significance of the NACHT domain and its constituent motifs for coordinating global protein structure, inflammasome organization and downstream signaling events. The Walker A and Walker B motifs are deemed essential for ATP-binding and hydrolysis properties, respectively. However, other motifs (i.e., Sensor 1, Sensor 2, HD1 and WHD) are now known to contribute substantionally to the topology of the nucleotide-binding site. Advances in structural models and mechanistic biochemistry indicate that large-scale conformational changes to the basal, ADP-bound NACHT domain are induced by ATP binding and are transmitted globally to disengage autoinhibition and render NLRs available for oligomerization. The instigating factor for ATP binding is still unknown. Future studies should survey potential triggers for nucleotide exchange, and investigate candidates such as changes in post-translational modifications, removal of inhibitory protein-protein interactions, or cofactor binding.

Regrettably, many assumptions regarding the biochemistry of the NLRs are still based primarily on analogy with limited corroborating evidence. It is clear that additional holistic studies of the entire NLR family are required to address this knowledge deficit. Indeed, systems for the effective production of recombinant proteins are required, biochemical assessments of enzymatic function should be completed, and precise structural definitions of the catalytic NACHT domain are necessary. Creative application of novel technologies and reagents for biological interrogations will continue to advance understanding of the different NLR members. These data will be key to defining how the ATP-binding and hydrolysis properties of NACHT domains in different NLRs integrate with signaling modules and binding partners to control innate immune responses at the molecular level.

ATP hydrolysis is the primary enzymatic function of the NLRs, and concerted efforts are now being made to specifically target inhibitor development toward the nucleotide-binding properties of the NACHT domain. Moreover, insights provided by the mechanistic analyses of novel small molecule inhibitors continue to provide critical information on the mechanism of ATP hydrolysis and NLR activation, as a whole. The severity and considerable quantity of human diseases associated with aberrant NLRP3 signaling are clear motivations for the identification and refinement of specific and potent NLRP3 inhibitors. Historically, the development and biochemical characterization of NLRP3 antagonists has been hindered by a lack of detailed structural information and a precisely-characterized activation mechanism. Future drug discovery work seeking to target NLRP3 should combine computational and biochemical methodologies with the recent structure of the NLRP3-NEK7 complex for a more comprehensive approach to the development of innovative therapeutics.

## Supplementary Materials

The following are available online, Methods S1: Molecular Dynamic Simulations; Video S1: ADP-NLRP3 10 ns Molecular Dynamic Simulation; Video S2: ATP-NLRP3 10 ns Molecular Dynamic Simulation.

## References

[1] Vajjhala P.R., Ve T., Bentham A., Stacey K.J., Kobe B. (2017). The molecular mechanisms of signaling by cooperative assembly formation in innate immunity pathways. Mol. Immunol..

[2] Franz K.M., Kagan J.C. (2017). Innate immune receptors as competitive determinants of cell fate. Mol. Cell.

[3] Martinon F., Mayor A., Tschopp J. (2009). The inflammasomes: Guardians of the body. Ann. Rev. Immunol..

[4] Latz E., Xiao T.S., Stutz A. (2013). Activation and regulation of the inflammasomes. Nat. Rev. Immunol..

[5] Sharma D., Kanneganti T.D. (2016). The cell biology of inflammasomes: Mechanisms of inflammasome activation and regulation. J. Cell Biol..

[6] Bergsbaken T., Fink S.L., Cookson B.T. (2009). Pyroptosis: Host cell death and inflammation. Nat. Rev. Microbiol..

[7] Man S.M., Karki R., Kanneganti T.D. (2017). Molecular mechanisms and functions of pyroptosis, inflammatory caspases and inflammasomes in infectious diseases. Immunol. Rev..

[8] Sborgi L., Rühl S., Mulvihill E., Pipercevic J., Heilig R., Stahlberg H., Farady C.J., Müller D.J., Broz P., Hiller S. (2016). GSDMD membrane pore formation constitutes the mechanism of pyroptotic cell death. EMBO J..

[9] Platnich J.M., Muruve D.A. (2019). NOD-like receptors and inflammasomes: A review of their canonical and non-canonical signaling pathways. Arch. Biochem. Biophys..

[10] Ting J.P.Y., Lovering R.C., Alnemri E.S., Bertin J., Boss J.M., Davis B.K., Flavell R.A., Girardin S.E., Godzik A., Harton J.A. (2008). The NLR gene family: A standard nomenclature. Immunity.

[11] Koonin E.V., Aravind L. (2000). The NACHT family-A new group of predicted NTPases implicated in apoptosis and MHC transcription activation. Trends Biochem. Sci..

[12] Danot O., Marquenet E., Vidal-Ingigliardi D., Richet E. (2009). Wheel of life, wheel of death: A mechanistic insight into signaling by STAND proteins. Structure.

[13] Leipe D.D., Koonin E.V., Aravind L. (2004). STAND, a class of P-loop NTPases including animal and plant regulators of programmed cell death: Multiple, complex domain architectures, unusual phyletic patterns, and evolution by horizontal gene transfer. J. Mol. Biol..

[14] Arya P., Acharya V. (2016). Computational identification raises a riddle for distribution of putative NACHT NTPases in the genome of early green plants. PLoS ONE.

[15] Hu Z., Yan C., Liu P., Huang Z., Ma R., Zhang C., Wang R., Zhang Y., Martinon F., Miao D. (2013). Crystal structure of NLRC4 reveals its autoinhibition mechanism. Science.

[16] Waterhouse A.M., Procter J.B., Martin D.M.A., Clamp M., Barton G.J. (2009). Jalview Version 2-A multiple sequence alignment editor and analysis workbench. Bioinformatics.

[17] Edgar R.C. (2004). MUSCLE: Multiple sequence alignment with high accuracy and high throughput. Nucleic Acids Res..

[18] Crooks G.E., Hon G., Chandonia J.M., Brenner S.E. (2004). WebLogo: A sequence logo generator. Genome Res..

[19] Mathur A., Hayward J.A., Man S.M. (2018). Molecular mechanisms of inflammasome signaling. J. Leukoc. Biol..

[20] Lechtenberg B.C., Mace P.D., Riedl S.J. (2014). Structural mechanisms in NLR inflammasome signaling. Curr. Opin. Struct. Biol..

[21] Hauenstein A.V., Zhang L., Wu H. (2015). The hierarchical structural architecture of inflammasomes, supramolecular inflammatory machines. Curr. Opin. Struct. Biol..

[22] Boucher D., Monteleone M., Coll R.C., Chen K.W., Ross C.M., Teo J.L., Gomez G.A., Holley C.L., Bierschenk D., Stacey K.J. (2018). Caspase-1 self-cleavage is an intrinsic mechanism to terminate inflammasome activity. J. Exp. Med..

[23] Strowig T., Henao-Mejia J., Elinav E., Flavell R. (2012). Inflammasomes in health and disease. Nature.

[24] Wang W., Zhang Y., Yang L., Li H. (2017). The innate immune signaling in cancer and cardiometabolic diseases: Friends or foes?. Cancer Lett..

[25] Conforti-Andreoni C., Ricciardi-Castagnoli P., Mortellaro A. (2011). The inflammasomes in health and disease: From genetics to molecular mechanisms of autoinflammation and beyond. Cell. Mol. Immunol..

[26] Hutton H.L., Ooi J.D., Holdsworth S.R., Kitching A.R. (2016). The NLRP3 inflammasome in kidney disease and autoimmunity. Nephrology.

[27] Kim Y.K., Shin J.S., Nahm M.H. (2016). NOD-like receptors in infection, immunity, and diseases. Yonsei Med. J..

[28] Guo H., Callaway J.B., Ting J.P.Y. (2015). Inflammasomes: Mechanism of action, role in disease, and therapeutics. Nat. Med..

[29] Christgen S., Kanneganti T.D. (2020). Inflammasomes and the fine line between defense and disease. Curr. Opin. Immunol..

[30] Martinon F., Burns K., Tschopp J. (2002). The Inflammasome: A molecular platform triggering activation of inflammatory caspases and processing of proIL-β. Mol. Cell.

[31] de Alba E. (2019). Structure, interactions and self-assembly of ASC-dependent inflammasomes. Arch. Biochem. Biophys..

[32] Sandstrom A., Mitchell P.S., Goers L., Mu E.W., Lesser C.F., Vance R.E. (2019). Functional degradation: A mechanism of NLRP1 inflammasome activation by diverse pathogen enzymes. Science.

[33] Finger J.N., Lich J.D., Dare L.C., Cook M.N., Brown K.K., Duraiswamis C., Bertin J.J., Gough P.J. (2012). Autolytic proteolysis within the function to find domain (FIIND) is required for NLRP1 inflammasome activity. J. Biol. Chem..

[34] Frew B.C., Joag V.R., Mogridge J. (2012). Proteolytic processing of Nlrp1b is required for inflammasome activity. PLoS Pathog..

[35] Faustin B., Lartigue L., Bruey J.M., Luciano F., Sergienko E., Bailly-Maitre B., Volkmann N., Hanein D., Rouiller I., Reed J.C. (2007). Reconstituted NALP1 inflammasome reveals two-step mechanism of caspase-1 activation. Mol. Cell.

[36] Poyet J.L., Srinivasula S.M., Tnani M., Razmara M., Fernandes-Alnemri T., Alnemri E.S. (2001). Identification of Ipaf, a human caspase-1-activating protein related to Apaf-1. J. Biol. Chem..

[37] Nour A.M., Yeung Y.G., Santambrogio L., Boyden E.D., Stanley E.R., Brojatsch J. (2009). Anthrax lethal toxin triggers the formation of a membrane-associated inflammasome complex in murine macrophages. Infect. Immun..

[38] Broz P., Dixit V.M. (2016). Inflammasomes: Mechanism of assembly, regulation and signalling. Nat. Rev. Immunol..

[39] Manji G.A., Wang L., Geddes B.J., Brown M., Merriam S., Al-Garawi A., Mak S., Lora J.M., Briskin M., Jurman M. (2002). PYPAF1, a PYRIN-containing Apaf1-like protein that assembles with ASC and regulates activation of NF-κB. J. Biol. Chem..

[40] Yang X., Lin G., Han Z., Chai J. (2019). Structural biology of NOD-like receptors. Advances in Experimental Medicine and Biology.

[41] Duncan J.A., Bergstralh D.T., Wang Y., Willingham S.B., Ye Z., Zimmermann A.G., Ting J.P.Y. (2007). Cryopyrin/NALP3 binds ATP/dATP, is an ATPase, and requires ATP binding to mediate inflammatory signaling. Proc. Natl. Acad. Sci. USA.

[42] Ye Z., Lich J.D., Moore C.B., Duncan J.A., Williams K.L., Ting J.P.-Y. (2008). ATP binding by Monarch-1/NLRP12 Is critical for its inhibitory function. Mol. Cell. Biol..

[43] Radian A.D., Khare S., Chu L.H., Dorfleutner A., Stehlik C. (2015). ATP binding by NLRP7 is required for inflammasome activation in response to bacterial lipopeptides. Mol. Immunol..

[44] Martino L., Holland L., Christodoulou E., Kunzelmann S., Esposito D., Rittinger K. (2016). The biophysical characterisation and SAXS analysis of human NLRP1 uncover a new level of complexity of NLR proteins. PLoS ONE.

[45] Zurek B., Proell M., Wagner R.N., Schwarzenbacher R., Kufer T.A. (2012). Mutational analysis of human NOD1 and NOD2 NACHT domains reveals different modes of activation. Innate Immun..

[46] Maharana J., Sahoo B.R., Bej A., Jena I., Parida A., Sahoo J.R., Dehury B., Patra M.C., Martha S.R., Balabantray S. (2015). Structural models of zebrafish (Danio rerio) NOD1 and NOD2 NACHT domains suggest differential ATP binding orientations: Insights from computational modeling, docking and molecular dynamics simulations. PLoS ONE.

[47] Maharana J., Panda D., De S. (2018). Deciphering the ATP-binding mechanism(s) in NLRP-NACHT 3D models using structural bioinformatics approaches. PLoS ONE.

[48] Uhlén M., Fagerberg L., Hallström B.M., Lindskog C., Oksvold P., Mardinoglu A., Sivertsson Å., Kampf C., Sjöstedt E., Asplund A. (2015). Tissue-based map of the human proteome. Science.

[49] Ardlie K.G., DeLuca D.S., Segrè A.V., Sullivan T.J., Young T.R., Gelfand E.T., Trowbridge C.A., Maller J.B., Tukiainen T., Lek M. (2015). The Genotype-Tissue Expression (GTEx) pilot analysis: Multitissue gene regulation in humans. Science.

[50] Lizio M., Abugessaisa I., Noguchi S., Kondo A., Hasegawa A., Hon C.C., De Hoon M., Severin J., Oki S., Hayashizaki Y. (2019). Update of the FANTOM web resource: Expansion to provide additional transcriptome atlases. Nucleic Acids Res..

[51] Bahar Halpern K., Caspi I., Lemze D., Levy M., Landen S., Elinav E., Ulitsky I., Itzkovitz S. (2015). Nuclear Retention of mRNA in Mammalian Tissues. Cell Rep..

[52] van der Heijden C.D.C.C., Noz M.P., Joosten L.A.B., Netea M.G., Riksen N.P., Keating S.T. (2018). Epigenetics and trained immunity. Antioxid. Redox Signal..

[53] Netea M.G., Schlitzer A., Placek K., Joosten L.A.B., Schultze J.L. (2019). Innate and adaptive immune memory: Aan evolutionary continuum in the host’s response to pathogens. Cell Host Microbe.

[54] Chen S., Yang J., Wei Y., Wei X. (2020). Epigenetic regulation of macrophages: From homeostasis maintenance to host defense. Cell. Mol. Immunol..

[55] Mehta S., Jeffrey K.L. (2015). Beyond receptors and signaling: Epigenetic factors in the regulation of innate immunity. Immunol. Cell Biol..

[56] Kelly B., O’Neill L.A.J. (2015). Metabolic reprogramming in macrophages and dendritic cells in innate immunity. Cell Res..

[57] Jin Y., Fu J. (2019). Novel Insights into the NLRP3 Inflammasome in atherosclerosis. J. Am. Heart Assoc..

[58] Christ A., Günther P., Lauterbach M.A.R., Duewell P., Biswas D., Pelka K., Scholz C.J., Oosting M., Haendler K., Baßler K. (2018). Western diet triggers NLRP3-dependent innate immune reprogramming. Cell.

[59] Ciraci C., Janczy J.R., Sutterwala F.S., Cassel S.L. (2012). Control of innate and adaptive immunity by the inflammasome. Microbes Infect..

[60] Evavold C.L., Kagan J.C. (2018). How inflammasomes inform adaptive immunity. J. Mol. Biol..

[61] Trunk G., Oxenius A. (2012). Innate instruction of CD4+ T cell immunity in respiratory bacterial infection. J. Immunol..

[62] Pedra J.H.F., Sutterwala F.S., Sukumaran B., Ogura Y., Qian F., Montgomery R.R., Flavell R.A., Fikrig E. (2007). ASC/PYCARD and caspase-1 regulate the IL-18/IFN-γ axis during Anaplasma phagocytophilum infection. J. Immunol..

[63] Ichinohe T., Lee H.K., Ogura Y., Flavell R., Iwasaki A. (2009). Inflammasome recognition of influenza virus is essential for adaptive immune responses. J. Exp. Med..

[64] Ghiringhelli F., Apetoh L., Tesniere A., Aymeric L., Ma Y., Ortiz C., Vermaelen K., Panaretakis T., Mignot G., Ullrich E. (2009). Activation of the NLRP3 inflammasome in dendritic cells induces IL-1Β-dependent adaptive immunity against tumors. Nat. Med..

[65] Felley L.E., Sharma A., Theisen E., Romero-Masters J.C., Sauer J.-D., Gumperz J.E. (2016). Human invariant NKT cells induce IL-1β secretion by peripheral blood monocytes via a P2X7-independent pathway. J. Immunol..

[66] Guarda G., Dostert C., Staehli F., Cabalzar K., Castillo R., Tardivel A., Schneider P., Tschopp J. (2009). T cells dampen innate immune responses through inhibition of NLRP1 and NLRP3 inflammasomes. Nature.

[67] Meyers B.C., Dickerman A.W., Michelmore R.W., Sivaramakrishnan S., Sobral B.W., Young N.D. (1999). Plant disease resistance genes encode members of an ancient and diverse protein family within the nucleotide-binding superfamily. Plant J..

[68] Tameling W.I.L., Elzinga S.D.J., Darmin P.S., Vossen J.H., Takken F.L.W., Haring M.A., Cornelissen B.J.C. (2002). The tomato R gene products i-2 and Mi-1 are functional ATP binding proteins with ATPase activity. Plant Cell.

[69] Chinnaiyan A., Chaudhary D., O’Rourke K., Koonin E.V., Dixit V.M. (1997). Role of CED-4 in the activation of CED-3. Nature.

[70] Zou H., Li Y., Liu X., Wang X. (1999). An APAf-1 · cytochrome C multimeric complex is a functional apoptosome that activates procaspase-9. J. Biol. Chem..

[71] Jiang X., Wang X. (2000). Cytochrome c promotes caspase-9 activation by inducing nucleotide binding to Apaf-1. J. Biol. Chem..

[72] van der Biezen E.A., Jones J.D. (1998). The NB-ARC domain: A novel signalling motif shared by plant resistance gene products and regulators of cell death in animals. Curr. Biol..

[73] Ammelburg M., Frickey T., Lupas A.N. (2006). Classification of AAA+ proteins. J. Struct. Biol..

[74] Dorstyn L., Akey C.W., Kumar S. (2018). New insights into apoptosome structure and function. Cell Death Differ..

[75] Fusco W.G., Duncan J.A. (2018). Novel aspects of the assembly and activation of inflammasomes with focus on the NLRC4 inflammasome. Int. Immunol..

[76] Sharif H., Wang L., Wang W.L., Magupalli V.G., Andreeva L., Qiao Q., Hauenstein A.V., Wu Z., Núñez G., Mao Y. (2019). Structural mechanism for NEK7-licensed activation of NLRP3 inflammasome. Nature.

[77] Hughes A.L. (2006). Evolutionary relationships of vertebrate NACHT domain-containing proteins. Immunogenetics.

[78] Proell M., Riedl S.J., Fritz J.H., Rojas A.M., Schwarzenbacher R. (2008). The Nod-Like Receptor (NLR) family: A tale of similarities and differences. PLoS ONE.

[79] Tian X., Pascal G., Monget P. (2009). Evolution and functional divergence of NLRP genes in mammalian reproductive systems. BMC Evol. Biol..

[80] Liao K.C., Sandall C.F., Carlson D.A., Ulke-Lemée A., Platnich J.M., Hughes P.F., Muruve D.A., Haystead T.A.J., MacDonald J.A. (2019). Application of immobilized ATP to the study of NLRP inflammasomes. Arch. Biochem. Biophys..

[81] Yan N., Chai J., Eui S.L., Gu L., Liu Q., He J., Wu J.W., Kokel D., Li H., Hao Q. (2005). Structure of the CED-4-CED-9 complex provides insights into programmed cell death in Caenorhabditis elegans. Nature.

[82] Murphy J.M., Farhan H., Eyers P.A. (2017). Bio-Zombie: The rise of pseudoenzymes in biology. Biochem. Soc. Trans..

[83] Murphy J.M., Mace P.D., Eyers P.A. (2017). Live and let die: Insights into pseudoenzyme mechanisms from structure. Curr. Opin. Struct. Biol..

[84] MacDonald J.A., Wijekoon C.P., Liao K.C., Muruve D.A. (2013). Biochemical and structural aspects of the ATP-binding domain in inflammasome-forming human NLRP proteins. IUBMB Life.

[85] Sandall C.F., MacDonald J.A. (2019). Effects of phosphorylation on the NLRP3 inflammasome. Arch. Biochem. Biophys..

[86] Duncan J.A., Canna S.W. (2018). The NLRC4 Inflammasome. Immunol. Rev..

[87] Kofoed E.M., Vance R.E. (2011). Innate immune recognition of bacterial ligands by NAIPs determines inflammasome specificity. Nature.

[88] Zhao Y., Yang J., Shi J., Gong Y.N., Lu Q., Xu H., Liu L., Shao F. (2011). The NLRC4 inflammasome receptors for bacterial flagellin and type III secretion apparatus. Nature.

[89] Lightfield K.L., Persson J., Trinidad N.J., Brubaker S.W., Kofoed E.M., Sauer J.D., Dunipace E.A., Warren S.E., Miao E.A., Vance R.E. (2011). Differential requirements for NAIP5 in activation of the NLRC4 inflammasome. Infect. Immun..

[90] Miao E.A., Mao D.P., Yudkovsky N., Bonneau R., Lorang C.G., Warren S.E., Leaf I.A., Aderem A. (2010). Innate immune detection of the type III secretion apparatus through the NLRC4 inflammasome. Proc. Natl. Acad. Sci. USA.

[91] Tenthorey J.L., Kofoed E.M., Daugherty M.D., Malik H.S., Vance R.E. (2014). Molecular Basis for Specific Recognition of Bacterial Ligands by NAIP/NLRC4 Inflammasomes. Mol. Cell.

[92] Diebolder C.A., Halff E.F., Koster A.J., Huizinga E.G., Koning R.I. (2015). Cryoelectron Tomography of the NAIP5/NLRC4 Inflammasome: Implications for NLR Activation. Structure.

[93] Hu Z., Zhou Q., Zhang C., Fan S., Cheng W., Zhao Y., Shao F., Wang H.W., Sui S.F., Chai J. (2015). Structural and biochemical basis for induced self-propagation of NLRC4. Science.

[94] Zhang L., Chen S., Ruan J., Wu J., Tong A.B., Yin Q., Li Y., David L., Lu A., Wang W.L. (2015). Cryo-EM structure of the activated NAIP2-NLRC4 inflammasome reveals nucleated polymerization. Science.

[95] Li Y., Fu T.M., Lu A., Witt K., Ruan J., Shen C., Wu H. (2018). Cryo-EM structures of ASC and NLRC4 CARD filaments reveal a unified mechanism of nucleation and activation of caspase-1. Proc. Natl. Acad. Sci. USA.

[96] Lu C., Wang A., Wang L., Dorsch M., Ocain T.D., Xu Y. (2005). Nucleotide binding to CARD12 and its role in CARD12-mediated caspase-1 activation. Biochem. Biophys. Res. Commun..

[97] Canna S.W., De Jesus A.A., Gouni S., Brooks S.R., Marrero B., Liu Y., Dimattia M.A., Zaal K.J.M., Sanchez G.A.M., Kim H. (2014). An activating NLRC4 inflammasome mutation causes autoinflammation with recurrent macrophage activation syndrome. Nat. Genet..

[98] Romberg N., Al Moussawi K., Nelson-Williams C., Stiegler A.L., Loring E., Choi M., Overton J., Meffre E., Khokha M.K., Huttner A.J. (2014). Mutation of NLRC4 causes a syndrome of enterocolitis and autoinflammation. Nat. Genet..

[99] Mitchell P.S., Sandstrom A., Vance R.E. (2019). The NLRP1 inflammasome: New mechanistic insights and unresolved mysteries. Curr. Opin. Immunol..

[100] Taabazuing C.Y., Griswold A.R., Bachovchin D.A. (2020). The NLRP1 and CARD8 inflammasomes. Immunol. Rev..

[101] Harris P.A., Duraiswami C., Fisher D.T., Fornwald J., Hoffman S.J., Hofmann G., Jiang M., Lehr R., McCormick P.M., Nickels L. (2015). High throughput screening identifies ATP-competitive inhibitors of the NLRP1 inflammasome. Bioorganic Med. Chem. Lett..

[102] Liao K.C., Mogridge J. (2013). Activation of the Nlrp1b inflammasome by reduction of cytosolic ATP. Infect. Immun..

[103] Minkiewicz J., de Rivero Vaccari J.P., Keane R.W. (2013). Human astrocytes express a novel NLRP2 inflammasome. Glia.

[104] Matsuoka Y., Yamashita A., Matsuda M., Kawai K., Sawa T., Amaya F. (2019). NLRP2 inflammasome in dorsal root ganglion as a novel molecular platform that produces inflammatory pain hypersensitivity. Pain.

[105] Bruey J.M., Bruey-Sedano N., Newman R., Chandler S., Stehlik C., Reed J.C. (2004). PAN1/ NALP2/PYPAF2, an inducible inflammatory mediator that regulates NF-κB and caspase-1 activation in macrophages. J. Biol. Chem..

[106] Tilburgs T., Meissner T.B., Ferreira L.M.R., Mulder A., Musunuru K., Ye J., Strominger J.L. (2017). NLRP2 is a suppressor of NF-κB signaling and HLA-C expression in human trophoblasts. Biol. Reprod..

[107] Rossi M.N., Pascarella A., Licursi V., Caiello I., Taranta A., Rega L.R., Levtchenko E., Emma F., De Benedetti F., Prencipe G. (2019). NLRP2 Regulates Proinflammatory and Antiapoptotic Responses in Proximal Tubular Epithelial Cells. Front. Cell Dev. Biol..

[108] Peng H., Chang B., Lu C., Su J., Wu Y., Lv P., Wang Y., Liu J., Zhang B., Quan F. (2012). Nlrp2, a maternal effect gene required for early embryonic development in the mouse. PLoS ONE.

[109] Kuchmiy A.A., D’Hont J., Hochepied T., Lamkanfi M. (2016). NLRP2 controls age-associated maternal fertility. J. Exp. Med..

[110] Peng H., Liu H., Liu F., Gao Y., Chen J., Huo J., Han J., Xiao T., Zhang W. (2017). NLRP2 and FAF1 deficiency blocks early embryogenesis in the mouse. Reproduction.

[111] Mahadevan S., Sathappan V., Utama B., Lorenzo I., Kaskar K., Van Den Veyver I.B. (2017). Maternally expressed NLRP2 links the subcortical maternal complex (SCMC) to fertility, embryogenesis and epigenetic reprogramming. Sci. Rep..

[112] Mu J., Wang W., Chen B., Wu L., Li B., Mao X., Zhang Z., Fu J., Kuang Y., Sun X. (2019). Mutations in NLRP2 and NLRP5 cause female infertility characterised by early embryonic arrest. J. Med. Genet..

[113] Agostini L., Martinon F., Burns K., McDermott M.F., Hawkins P.N., Tschopp J. (2004). NALP3 forms an IL-1β-processing inflammasome with increased activity in Muckle-Wells autoinflammatory disorder. Immunity.

[114] Fontalba A., Gutierrez O., Fernandez-Luna J.L. (2007). NLRP2, an inhibitor of the NF-κB pathway, is transcriptionally activated by NF-κB and exhibits a nonfunctional allelic variant. J. Immunol..

[115] Sutterwala F.S., Haasken S., Cassel S.L. (2014). Mechanism of NLRP3 inflammasome activation. Ann. N. Y. Acad. Sci..

[116] Harijith A., Ebenezer D.L., Natarajan V. (2014). Reactive oxygen species at the crossroads of inflammasome and inflammation. Front. Physiol..

[117] Freeman T.L., Swartz T.H. (2020). Targeting the NLRP3 Inflammasome in Severe COVID-19. Front. Immunol..

[118] van den Berg D.F., te Velde A.A. (2020). Severe COVID-19: NLRP3 Inflammasome Dysregulated. Front. Immunol..

[119] Deftereos S.G., Siasos G., Giannopoulos G., Vrachatis D.A., Angelidis C., Giotaki S.G., Gargalianos P., Giamarellou H., Gogos C., Daikos G. (2020). The Greek study in the effects of colchicine in Covid-19 complications prevention (GRECCO-19 study): Rationale and study design. Hell. J. Cardiol..

[120] Chen J., Chen Z.J. (2018). PtdIns4P on dispersed trans-Golgi network mediates NLRP3 inflammasome activation. Nature.

[121] Zhang Z., Meszaros G., He W.T., Xu Y., de Magliarelli H.F., Mailly L., Mihlan M., Liu Y., Gámez M.P., Goginashvili A. (2017). Protein kinase D at the Golgi controls NLRP3 inflammasome activation. J. Exp. Med..

[122] Mortimer L., Moreau F., MacDonald J.A., Chadee K. (2016). NLRP3 inflammasome inhibition is disrupted in a group of auto-inflammatory disease CAPS mutations. Nat. Immunol..

[123] Guo C., Xie S., Chi Z., Zhang J., Liu Y., Zhang L., Zheng M., Zhang X., Xia D., Ke Y. (2016). Bile Acids Control Inflammation and Metabolic Disorder through Inhibition of NLRP3 Inflammasome. Immunity.

[124] Schmid-Burgk J.L., Chauhan D., Schmidt T., Ebert T.S., Reinhardt J., Endl E., Hornung V. (2016). A genome-wide CRISPR (clustered regularly interspaced short palindromic repeats) screen identifies NEK7 as an essential component of NLRP3 inflammasome activation. J. Biol. Chem..

[125] He Y., Zeng M.Y., Yang D., Motro B., Núñez G. (2016). NEK7 is an essential mediator of NLRP3 activation downstream of potassium efflux. Nature.

[126] Shi H., Wang Y., Li X., Zhan X., Tang M., Fina M., Su L., Pratt D., Hui Bu C., Hildebrand S. (2016). NLRP3 activation and mitosis are mutually exclusive events coordinated by NEK7, a new inflammasome component. Nat. Immunol..

[127] Tartey S., Kanneganti T.D. (2020). Inflammasomes in the pathophysiology of autoinflammatory syndromes. J. Leukoc. Biol..

[128] Touitou I., Lesage S., McDermott M., Cuisset L., Hoffman H., Dode C., Shoham N., Aganna E., Hugot J.P., Wise C. (2004). Infevers: An evolving mutation database for auto-inflammatory syndromes. Hum. Mutat..

[129] Meng G., Strober W. (2010). New insights into the nature of autoinflammatory diseases from mice with Nlrp3 mutations. Eur. J. Immunol..

[130] Anand P.K., Kanneganti T.D. (2013). NLRP6 in infection and inflammation. Microbes Infect..

[131] Levy M., Shapiro H., Thaiss C.A., Elinav E. (2017). NLRP6: A Multifaceted Innate Immune Sensor. Trends Immunol..

[132] Ghimire L., Paudel S., Jin L., Jeyaseelan S. (2020). The NLRP6 inflammasome in health and disease. Mucosal Immunol..

[133] Wang P., Zhu S., Yang L., Cui S., Pan W., Jackson R., Zheng Y., Rongvaux A., Sun Q., Yang G. (2015). Nlrp6 regulates intestinal antiviral innate immunity. Science..

[134] Levy M., Thaiss C.A., Zeevi D., Dohnalová L., Zilberman-Schapira G., Mahdi J.A., David E., Savidor A., Korem T., Herzig Y. (2015). Microbiota-modulated metabolites shape the intestinal microenvironment by regulating NLRP6 inflammasome signaling. Cell.

[135] Seregin S.S., Golovchenko N., Schaf B., Chen J., Eaton K.A., Chen G.Y. (2017). NLRP6 function in inflammatory monocytes reduces susceptibility to chemically induced intestinal injury. Mucosal Immunol..

[136] Anand P.K., Subbarao Malireddi R.K., Lukens J.R., Vogel P., Bertin J., Lamkanfi M., Kanneganti T.D. (2012). NLRP6 negatively regulates innate immunity and host defence against bacterial pathogens. Nature.

[137] Wlodarska M., Thaiss C.A., Nowarski R., Henao-Mejia J., Zhang J.P., Brown E.M., Frankel G., Levy M., Katz M.N., Philbrick W.M. (2014). NLRP6 inflammasome orchestrates the colonic host-microbial interface by regulating goblet cell mucus secretion. Cell.

[138] Shen C., Lu A., Xie W.J., Ruan J., Negro R., Egelman E.H., Fu T.M., Wu H. (2019). Molecular mechanism for NLRP6 inflammasome assembly and activation. Proc. Natl. Acad. Sci. USA.

[139] Leng F., Yin H., Qin S., Zhang K., Guan Y., Fang R., Wang H., Li G., Jiang Z., Sun F. (2020). NLRP6 self-assembles into a linear molecular platform following LPS binding and ATP stimulation. Sci. Rep..

[140] Lu A., Magupalli V.G., Ruan J., Yin Q., Atianand M.K., Vos M.R., Schröder G.F., Fitzgerald K.A., Wu H., Egelman E.H. (2014). Unified polymerization mechanism for the assembly of asc-dependent inflammasomes. Cell.

[141] Khare S., Dorfleutner A., Bryan N.B., Yun C., Radian A.D., de Almeida L., Rojanasakul Y., Stehlik C. (2012). An NLRP7-Containing Inflammasome Mediates Recognition of Microbial Lipopeptides in Human Macrophages. Immunity.

[142] Zhou Y., Shah S.Z.A., Yang L., Zhang Z., Zhou X., Zhao D. (2016). Virulent Mycobacterium bovis Beijing strain activates the NLRP7 inflammasome in THP-1 macrophages. PLoS ONE.

[143] Pinheiro A.S., Proell M., Eibl C., Page R., Schwarzenbacher R., Peti W. (2010). Three-dimensional structure of the NLRP7 pyrin domain insight into pyrin-pyrin-mediated effector domain signaling in innate immunity. J. Biol. Chem..

[144] Singer H., Biswas A., Zimmer N., Messaed C., Oldenburg J., Slim R., El-Maarri O. (2014). NLRP7 inter-domain interactions: The NACHT-associated domain is the physical mediator for oligomeric assembly. Mol. Hum. Reprod..

[145] Zhu S., Ding S., Wang P., Wei Z., Pan W., Palm N.W., Yang Y., Yu H., Li H.B., Wang G. (2017). Nlrp9b inflammasome restricts rotavirus infection in intestinal epithelial cells. Nature.

[146] Ha H.J., Park H.H. (2020). Crystal structure of the human NLRP9 pyrin domain reveals a bent N-terminal loop that may regulate inflammasome assembly. FEBS Lett..

[147] Marleaux M., Anand K., Latz E., Geyer M. (2020). Crystal structure of the human NLRP9 pyrin domain suggests a distinct mode of inflammasome assembly. FEBS Lett..

[148] Wang L., Manji G.A., Grenier J.M., Al-Garawi A., Merriam S., Lora J.M., Geddes B.J., Briskin M., DiStefano P.S., Bertin J. (2002). PYPAF7, a novel PYRIN-containing Apaf1-like protein that regulates activation of NF-kappa B and caspase-1-dependent cytokine processing. J. Biol. Chem..

[149] Vladimer G.I., Weng D., Paquette S.W.M., Vanaja S.K., Rathinam V.A.K., Aune M.H., Conlon J.E., Burbage J.J., Proulx M.K., Liu Q. (2012). The NLRP12 Inflammasome Recognizes Yersinia pestis. Immunity.

[150] Tuncer S., Fiorillo M.T., Sorrentino R. (2014). The multifaceted nature of NLRP12. J. Leukoc. Biol..

[151] Jéru I., Le Borgne G., Cochet E., Hayrapetyan H., Duquesnoy P., Grateau G., Morali A., Sarkisian T., Amselem S. (2011). Identification and functional consequences of a recurrent NLRP12 missense mutation in periodic fever syndromes. Arthritis Rheum..

[152] Jéru I., Duquesnoy P., Fernandes-Alnemri T., Cochet E., Yu J.W., Lackmy-Port-Lis M., Grimprel E., Landman-Parker J., Hentgen V., Marlin S. (2008). Mutations in NALP12 cause hereditary periodic fever syndromes. Proc. Natl. Acad. Sci. USA.

[153] Iyer L.M., Leipe D.D., Koonin E.V., Aravind L. (2004). Evolutionary history and higher order classification of AAA+ ATPases. J. Struct. Biol..

[154] Hanson P.I., Whiteheart S.W. (2005). AAA+ proteins: Have engine, will work. Nat. Rev. Mol. Cell Biol..

[155] Ogura T., Whiteheart S.W., Wilkinson A.J. (2004). Conserved arginine residues implicated in ATP hydrolysis, nucleotide-sensing, and inter-subunit interactions in AAA and AAA+ ATPases. J. Struct. Biol..

[156] Yang X., Yang F., Wang W., Lin G., Hu Z., Han Z., Qi Y., Zhang L., Wang J., Sui S.F. (2018). Structural basis for specific flagellin recognition by the NLR protein NAIP5. Cell Res..

[157] Davoodi J., Lin L., Kelly J., Liston P., MacKenzie A.E. (2004). Neuronal apoptosis-inhibitory protein does not interact with Smac and requires ATP to bind caspase-9. J. Biol. Chem..

[158] Karimpour S., Davoodi J., Ghahremani M.H. (2011). Integrity of ATP binding site is essential for effective inhibition of the intrinsic apoptosis pathway by NAIP. Biochem. Biophys. Res. Commun..

[159] Cai X., Chen J., Xu H., Liu S., Jiang Q.X., Halfmann R., Chen Z.J. (2014). Prion-like polymerization underlies signal transduction in antiviral immune defense and inflammasome activation. Cell.

[160] Sahillioglu A.C., Sumbul F., Ozoren N., Haliloglu T. (2014). Structural and dynamics aspects of ASC speck assembly. Structure.

[161] Lu A., Li Y., Yin Q., Ruan J., Yu X., Egelman E., Wu H. (2015). Plasticity in PYD assembly revealed by cryo-EM structure of the PYD filament of AIM2. Cell Discov..

[162] Lu A., Li Y., Schmidt F.I., Yin Q., Chen S., Fu T.M., Tong A.B., Ploegh H.L., Mao Y., Wu H. (2016). Molecular basis of caspase-1 polymerization and its inhibition by a new capping mechanism. Nat. Struct. Mol. Biol..

[163] Maharana J., Dehury B., Sahoo J.R., Jena I., Bej A., Panda D., Sahoo B.R., Patra M.C., Pradhan S.K. (2015). Structural and functional insights into CARDs of zebrafish (Danio rerio) NOD1 and NOD2, and their interaction with adaptor protein RIP2. Mol. Biosyst..

[164] Maharana J., Pradhan S.K., De S. (2017). NOD1CARD might be using multiple interfaces for RIP2-mediated CARD-CARD interaction: Insights from molecular dynamics simulation. PLoS ONE.

[165] Maharana J., Patra M.C., De B.C., Sahoo B.R., Behera B.K., De S., Pradhan S.K. (2014). Structural insights into the MDP binding and CARD-CARD interaction in zebrafish (Danio rerio) NOD2: A molecular dynamics approach. J. Mol. Recognit..

[166] Maharana J., Vats A., Gautam S., Nayak B.P., Kumar S., Sendha J., De S. (2017). POP1 might be recruiting its type-Ia interface for NLRP3-mediated PYD-PYD interaction: Insights from MD simulation. J. Mol. Recognit..

[167] Maharana J. (2018). Elucidating the interfaces involved in CARD-CARD interactions mediated by NLRP1 and Caspase-1 using molecular dynamics simulation. J. Mol. Graph. Model..

[168] Huber R.G., Eibl C., Fuchs J.E. (2015). Intrinsic flexibility of NLRP pyrin domains is a key factor in their conformational dynamics, fold stability, and dimerization. Protein Sci..

[169] Reubold T.F., Wohlgemuth S., Eschenburg S. (2011). Crystal structure of full-length Apaf-1: How the death signal is relayed in the mitochondrial pathway of apoptosis. Structure.

[170] Yuan S., Topf M., Reubold T.F., Eschenburg S., Akey C.W. (2013). Changes in Apaf-1 conformation that drive apoptosome assembly. Biochemistry.

[171] Halff E.F., Diebolder C.A., Versteeg M., Schouten A., Brondijk T.H.C., Huizinga E.G. (2012). Formation and structure of a NAIP5-NLRC4 inflammasome induced by direct interactions with conserved N- and C-terminal regions of flagellin. J. Biol. Chem..

[172] Reubold T.F., Wohlgemuth S., Eschenburg S. (2009). A new model for the transition of APAF-1 from inactive monomer to caspase-activating apoptosome. J. Biol. Chem..

[173] Afanasyeva A., Hirtreiter A., Schreiber A., Grohmann D., Pobegalov G., McKay A.R., Tsaneva I., Petukhov M., Käs E., Grigoriev M. (2014). Lytic water dynamics reveal evolutionarily conserved mechanisms of ATP hydrolysis by TIP49 AAA+ ATPases. Structure.

[174] Maekawa S., Ohto U., Shibata T., Miyake K., Shimizu T. (2016). Crystal structure of NOD2 and its implications in human disease. Nat. Commun..

[175] Tenthorey J.L., Haloupek N., López-Blanco J.R., Grob P., Adamson E., Hartenian E., Lind N.A., Bourgeois N.M., Chacón P., Nogales E. (2017). The structural basis of flagellin detection by NAIP5: A strategy to limit pathogen immune evasion. Science.

[176] Hafner-Bratkovič I., Sušjan P., Lainšček D., Tapia-Abellán A., Cerović K., Kadunc L., Angosto-Bazarra D., Pelegrίn P., Jerala R. (2018). NLRP3 lacking the leucine-rich repeat domain can be fully activated via the canonical inflammasome pathway. Nat. Commun..

[177] Dowds T.A., Masumoto J., Zhu L., Inohara N., Núñez G. (2004). Cryopyrin-induced interleukin 1β secretion in monocytic cells: Enhanced activity of disease-associated mutants and requirement for ASC. J. Biol. Chem..

[178] Martinon F., Agostini L., Meylan E., Tschopp J. (2004). Identification of bacterial muramyl dipeptide as activator of the NALP3/Cryopyrin inflammasome. Curr. Biol..

[179] Hayashi H., Cuddy M., Shu V.C.W., Yip K.W., Madiraju C., Diaz P., Matsuyama T., Kaibara M., Taniyama K., Vasile S. (2009). Versatile assays for high throughput screening for activatorsor inhibitors of intracellular proteases and their cellular regulators. PLoS ONE.

[180] Pettersen E.F., Goddard T.D., Huang C.C., Couch G.S., Greenblatt D.M., Meng E.C., Ferrin T.E. (2004). UCSF Chimera-A visualization system for exploratory research and analysis. J. Comput. Chem..

[181] Mark P., Nilsson L. (2001). Structure and dynamics of the TIP3P, SPC, and SPC/E water models at 298 K. J. Phys. Chem. A.

[182] Brooks B.R., Brooks C.L., Mackerell A.D., Nilsson L., Petrella R.J., Roux B., Won Y., Archontis G., Bartels C., Boresch S. (2009). CHARMM: The biomolecular simulation program. J. Comp. Chem..

[183] Vanommeslaeghe K., Hatcher E., Acharya C., Kundu S., Zhong S., Shim J., Darian E. (2010). CHARMM General Force Field: A force field for drug-like molecules compatible with the CHARMM all-atom additive biological force fields. J. Comput. Chem..

[184] DeLano W.L. (2002). Pymol: An Open-Source Molecular Graphics Tool. CCP4 Newsletter on Protein Crystallography.

[185] Humphrey W., Dalke A., Schulte K. (1996). VMD: Visual Molecular Dynamics. J. Mol. Graph..

[186] Yang Y., Wang H., Kouadir M., Song H., Shi F. (2019). Recent advances in the mechanisms of NLRP3 inflammasome activation and its inhibitors. Cell Death Dis..

[187] Zahid A., Li B., Kombe A.J.K., Jin T., Tao J. (2019). Pharmacological inhibitors of the NLRP3 inflammasome. Front. Immunol..

[188] Su M., Wang W., Liu F., Li H. (2020). Recent progress on the discovery of NLRP3 inhibitors and their therapeutic potential. Curr. Med. Chem..

[189] Perregaux D.G., McNiff P., Laliberte R., Hawryluk N., Peurano H., Stam E., Eggler J., Griffiths R., Dombroski M.A., Gabel C.A. (2001). Identification and characterization of a novel class of interleukin-1 post-translational processing inhibitors. J. Pharmacol. Exp. Ther..

[190] Lamkanfi M., Mueller J.L., Vitari A.C., Misaghi S., Fedorova A., Deshayes K., Lee W.P., Hoffman H.M., Dixit V.M. (2009). Glyburide inhibits the Cryopyrin/Nalp3 inflammasome. J. Cell Biol..

[191] Coll R.C., Robertson A.A., Chae J.J., Higgins S.C., Muñoz-Planillo R., Inserra M.C., Vetter I., Dungan L.S., Monks B.G., Stutz A. (2015). A small-molecule inhibitor of the NLRP3 inflammasome for the treatment of inflammatory diseases. Nat. Med..

[192] Coll R.C., Hill J.R., Day C.J., Zamoshnikova A., Boucher D., Massey N.L., Chitty J.L., Fraser J.A., Jennings M.P., Robertson A.A.B. (2019). MCC950 directly targets the NLRP3 ATP-hydrolysis motif for inflammasome inhibition. Nat. Chem. Biol..

[193] Tapia-Abellán A., Angosto-Bazarra D., Martínez-Banaclocha H., de Torre-Minguela C., Cerón-Carrasco J.P., Pérez-Sánchez H., Arostegui J.I., Pelegrin P. (2019). MCC950 closes the active conformation of NLRP3 to an inactive state. Nat. Chem. Biol..

[194] Groß C.J., Mishra R., Schneider K.S., Médard G., Wettmarshausen J., Dittlein D.C., Shi H., Gorka O., Koenig P.A., Fromm S. (2016). K+ Efflux-Independent NLRP3 Inflammasome Activation by Small Molecules Targeting Mitochondria. Immunity.

[195] Gaidt M.M., Ebert T.S., Chauhan D., Schmidt T., Schmid-Burgk J.L., Rapino F., Robertson A.A.B., Cooper M.A., Graf T., Hornung V. (2016). Human monocytes engage an alternative inflammasome pathway. Immunity.

[196] Juliana C., Fernandes-Alnemri T., Wu J., Datta P., Solorzano L., Yu J.-W., Meng R., Quang A.A., Latz E., Scott C.P. (2010). Anti-inflammatory compounds parthenolide and bay 11-7082 are direct inhibitors of the inflammasome. J. Biol. Chem..

[197] Lee J., Rhee M.H., Kim E., Cho J.Y. (2012). BAY 11-7082 is a broad-spectrum inhibitor with anti-inflammatory activity against multiple targets. Mediat. Inflamm..

[198] Jackson P.A., Widen J.C., Harki D.A., Brummond K.M. (2017). Covalent modifiers: A chemical perspective on the reactivity of α, β-unsaturated carbonyls with thiols via hetero-Michael addition reactions. J. Med. Chem..

[199] Kerr I.D., Lee J.J., Farady C.J., Marion R., Rickert M., Sajid M., Pandey K.C., Caffrey C.R., Legac J., Hansell E. (2009). Vinyl sulfones as antiparasitic agents and a structural basis for drug design. J. Biol. Chem..

[200] Juliana C., Fernandes-Alnemri T., Kang S., Farias A., Qin F., Alnemri E.S. (2012). Non-transcriptional priming and deubiquitination regulate NLRP3 inflammasome activation. J. Biol. Chem..

[201] Bertinaria M., Gastaldi S., Marini E., Giorgis M. (2019). Development of covalent NLRP3 inflammasome inhibitors: Chemistry and biological activity. Arch. Biochem. Biophys..

[202] Jiang H., He H., Chen Y., Huang W., Cheng J., Ye J., Wang A., Tao J., Wang C., Liu Q. (2017). Identification of a selective and direct NLRP3 inhibitor to treat inflammatory disorders. J. Exp. Med..

[203] Ma T., Thiagarajah J.R., Yang H., Sonawane N.D., Folli C., Galietta L.J.V., Verkman A.S. (2002). Thiazolidinone CFTR inhibitor identified by high-throughput screening blocks cholera toxin–induced intestinal fluid secretion. J. Clin. Invest..

[204] He Y., Varadarajan S., Mūnoz-Planillo Burberry A., Nakamura Y., Núñez G. (2014). 3,4-Methylenedioxy-β’-nitrostyrene inhibits NLRP3 inflammasome activation by blocking assembly of the inflammasome. J. Biol. Chem..

[205] Wang W.Y., Wu Y.C., Wu C.C. (2006). Prevention of platelet glycoprotein IIb/IIIa activation by 3,4-methylenedioxy-β-nitrostyrene, a novel tyrosine kinase inhibitor. Mol. Pharmacol..

[206] Marchetti C., Chojnacki J., Toldo S., Mezzaroma E., Tranchida N., Rose S.W., Federici M., Van Tassell B.W., Zhang S., Abbate A. (2014). A novel pharmacologic inhibitor of the NLRP3 inflammasome limits myocardial injury after ischemia-reperfusion in the mouse. J. Cardiovasc. Pharmacol..

[207] Marchetti C., Swartzwelter B., Koenders M.I., Azam T., Tengesdal I.W., Powers N., de Graff D.M., Dinarellow C.A., Joosten L.A.B. (2018). NLRP3 inflammasome inhibitor OLT1177 suppresses joint inflammation in murine models of acute arthritis. Arthritis Res. Ther..

[208] Marchetti C., Swartzwelter B., Gamboni F., Neff C.P., Richter K., Azam T., Carta S., Tengesdal I., Nemkov T., D’Alessandro A. (2018). OLT1177, a β-sulfonyl nitrile compound, safe in humans, inhibits the NLRP3 inflammasome and reverses the metabolic cost of inflammation. Proc. Natl. Acad. Sci. USA.

[209] Shim D.-W., Shin W.-Y., Yu S.-H., Kim B.-H., Ye S.-K., Koppula S., Won H.-S., Kang T.-B., Lee K.-H. (2017). BOT-4-one attenuates NLRP3 inflammasome activation: NLRP3 alkylation leading to the regulation of its ATPase activity and ubiquitination. Sci. Rep..

[210] Lee H.G., Cho N.-C., Jeong A.J., Li Y.-C., Rhie S.-J., Choi J.S., Lee K.-H., Kim Y., Kim Y.-N., Kim M.-H. (2016). Immunomodulatory activities of the benzoxathiole derivative BOT-4-one ameliorate pathogenic skin inflammation in mice. J. Invest. Dermatol..

[211] Kim B.-H., Yoon B.R., Kim E.K., Noh K.H., Kwon S.-H., Yi E.H., Lee H.G., Choi J.S., Kang S.W., Park I.-C. (2016). Alleviation of collagen-induced arthritis by the benzoxathiole derivative BOT-4-one in mice: Implication of the Th1-and Th17-cell-mediated immune responses. Biochem. Pharmacol..

[212] Cocco M., Garella D., Di Stilo A., Borretto E., Stevanato L., Giorgis M., Marini E., Fantozzi R., Miglio G., Bertinaria M. (2014). Electrophilic Warhead-Based Design of Compounds Preventing NLRP3 Inflammasome-Dependent Pyroptosis. J. Med. Chem..

[213] Cocco M., Pellegrini C., Martínez-Banaclocha H., Giorgis M., Marini E., Costale A., Miglio G., Fornai M., Antonioli L., López-Castejón G. (2017). Development of an Acrylate Derivative Targeting the NLRP3 Inflammasome for the Treatment of Inflammatory Bowel Disease. J. Med. Chem..

[214] Cocco M., Miglio G., Giorgis M., Garella D., Marini E., Costale A., Regazzoni L., Vistoli G., Orioli M., Massulaha-Ahmed R. (2016). Design, Synthesis, and Evaluation of Acrylamide Derivatives as Direct NLRP3 Inflammasome Inhibitors. ChemMedChem.

