# Supplementary material for: ATP-Binding and Hydrolysis in Inflammasome Activation
Source: Molecules. 2020 Oct 7;25(19):4572. doi: 10.3390/molecules25194572 (PMC7583971; doi:10.3390/molecules25194572)
Supplement: Supplementary file 1 [file molecules-25-04572-s001.zip › Supplemental/Methods S1 rev.docx]

# **Methods S1: Molecular Dynamic Simulations**

Molecular dynamic (MD) simulation of the NLRP3-ADP and ATP models was performed with the GROMACS 2020.2 package, using the CHARMM36 all-atom force field and TIP3P TIP 3-point water model [181,182]. MD simulations were run for 10 ns using GROMACS-2020.2 program. The starting structure of human NLRP3-ADP (PDB: 6NPY) was modeled directly (ADP-bound), or the ADP molecule was modified artificially in Chimera (ATP-bound). Ligand topology, atom typing and assignment of bonded parameters were performed with the CGenFF server [183]. The starting protein structures were immersed into a dodecahedron with boundaries extending 10 Å in all directions from the protein periphery and solvated with TIP3P water molecules. The charge of the system was neutralized with counter. The system was minimized using steepest descent algorithm until it converged with a maximum force no greater than 1000 kJ mol^−1^ nm^−1^. Temperature and pressure equilibration of the system was performed for 500 ps each, while restraining the ligand position, under NVT and NPT, respectively. Production runs were then performed upon the equilibrated systems for 10 ns at neutral pH, 300 K and 1 bar pressure, and snapshots of conformations were collected every 10 ps. Analyses of the trajectories were analysed using VMD software, UCSF Chimera and Pymol v2.4 [180,184-185].
